# Structural modelling and comparative analysis of homologous, analogous and specific proteins from *Trypanosoma cruzi *versus *Homo sapiens*: putative drug targets for chagas' disease treatment

**DOI:** 10.1186/1471-2164-11-610

**Published:** 2010-10-29

**Authors:** Priscila VSZ Capriles, Ana CR Guimarães, Thomas D Otto, Antonio B Miranda, Laurent E Dardenne, Wim M Degrave

**Affiliations:** 1 Grupo de Modelagem Molecular de Sistemas Biológicos, Laboratório Nacional de Computação Científica, LNCC/MCT, Petrópolis, CEP 25651-075, Brazil; 2 Laboratório de Genômica Funcional e Bioinformática, Instituto Oswaldo Cruz, IOC/FIOCRUZ, Rio de Janeiro, CEP 21045-900, Brazil; 3 Parasite Genomics, Wellcome Trust Sanger Institute, Wellcome Trust Genome Campus, Cambridge, CB10 1SA, UK; 4 Laboratório de Biologia Computacional e Sistemas, Instituto Oswaldo Cruz, IOC/FIOCRUZ, Rio de Janeiro, CEP 21045-900, Brazil

## Abstract

**Background:**

*Trypanosoma cruzi *is the etiological agent of Chagas' disease, an endemic infection that causes thousands of deaths every year in Latin America. Therapeutic options remain inefficient, demanding the search for new drugs and/or new molecular targets. Such efforts can focus on proteins that are specific to the parasite, but analogous enzymes and enzymes with a three-dimensional (3D) structure sufficiently different from the corresponding host proteins may represent equally interesting targets. In order to find these targets we used the workflows MHOLline and AnEnΠ obtaining 3D models from homologous, analogous and specific proteins of *Trypanosoma cruzi *versus *Homo sapiens*.

**Results:**

We applied genome wide comparative modelling techniques to obtain 3D models for 3,286 predicted proteins of *T*. *cruzi*. In combination with comparative genome analysis to *Homo sapiens*, we were able to identify a subset of 397 enzyme sequences, of which 356 are homologous, 3 analogous and 38 specific to the parasite.

**Conclusions:**

In this work, we present a set of 397 enzyme models of *T*. *cruzi *that can constitute potential structure-based drug targets to be investigated for the development of new strategies to fight Chagas' disease. The strategies presented here support the concept of structural analysis in conjunction with protein functional analysis as an interesting computational methodology to detect potential targets for structure-based rational drug design. For example, 2,4-dienoyl-CoA reductase (EC 1.3.1.34) and triacylglycerol lipase (EC 3.1.1.3), classified as analogous proteins in relation to *H. sapiens *enzymes, were identified as new potential molecular targets.

## Background

Chagas' disease constitutes a significant health and socio-economic problem in most of Central and South America and Mexico [1,2]. About 18 million people are infected resulting in an estimated 21,000 deaths per year (WHO, 2002). Cases have also been described in Canada, United States [3-5], Europe and Australia [6-8].

A hundred year after the discovery of Chagas' disease, caused by the haemoflagellate protozoan *Trypanosoma cruzi*, there are still no appropriate therapies that lead to consistent cure in the chronic phase of the disease. The importance of developing new, effective chemotherapies against Chagas' disease [9] is reinforced by its incidence death rate, the toxicity of the current drugs benznidazol and nifurtimox and the parasite's ability to develop drug resistance [10,11]. The analysis of the *T*. *cruzi *genome [12] opens new opportunities to develop more effective and less toxic drugs against the parasite.

Although therapeutic agents are also able to interact with polysaccharides, lipids and nucleic acids, protein inhibitors, particularly enzyme inhibitors, comprise about 47% of all drugs against pharmacological targets with commercial interest [13]. For this reason, this work is focused on enzymatic activities.

Metabolic pathways that are common to many diverse organisms are mostly made up of enzymatic reactions that are catalysed by conserved proteins. Enzymes which perform similar chemical reactions usually share similar structures, however analogous enzymes have little or no structural similarity, while sharing the same catalytic activity, and are thought to be evolutionarily unrelated [14]. *In silico *sequence analysis and comparisons of the primary and secondary structures *per se *cannot prove that two sequences are unrelated from an evolutionary point of view. A common origin can be inferred from protein structure conservation, even when evidence of homology at the amino acid level has been completely washed out by divergence. The possibility of a common origin can only be considered highly unlikely by additional confirmation that two proteins have different three-dimensional (3D) structures [15]. Furthermore, these differences of 3D structures are an important factor in selecting a protein as a potential therapeutic target [16].

During the process of the development of a new drug, many synthetic compounds or natural products are often tested. The efforts to isolate, purify, characterise, and synthesise active compounds and perform pre-and clinical tests take many years and can cost billions of dollars [17,18]. When an active compound is discovered, its mechanism of action is often unknown. Structure-based rational drug design intends to accelerate the steps of identification and comprehension of the molecular interactions between receptor and ligand using computational methods [19]. In this context, bioinformatics and molecular modelling tools can play an important role in the identification and structural investigation of molecular targets that are essential for the survival of *T*. *cruzi*. Indeed, candidate targets must be essential for the parasite's infectivity and/or survival, without affecting the (human) host [20]. Nonetheless, inhibitors should be efficient, soluble, bio-available and administrable in an acceptable way, having the potential for chemotherapeutic development [21].

Using comparative modelling techniques, it is possible to obtain protein models accurate enough to be used in structure-based rational drug design studies. Building models based on templates of homologous proteins that have had their 3D structure experimentally determined by X-Ray or Nuclear Magnetic Resonance has been useful for drug design, as they can guide the development of more specific non-natural inhibitors for variants of a given enzyme or receptor [22-24]. Conversely, models built based on low and medium similarity between the target and template sequences can be useful for functional inference, design of rational mutagenesis experiments and molecular replacement in crystallography. Thus, structural biology has been helpful in directing target identification and discovery, using high-throughput methods of structure determination, and providing an important tool for initial drug target screening and further optimisation [19].

A high-throughput functional genomics approach has been used to bridge the gap between raw genomic information and the identification of possible viable drug targets using techniques in biochemistry, molecular and cell biology, and bioinformatics [25]. This approach allows a better understanding of the role played by the steps in biological pathways involved in a variety of diseases.

The search for suitable targets for the development of new drugs in parasitosis is usually based on the identification of enzymes specific to the metabolic pathways of the parasite. However, data about the frequency and distribution of analogous enzymes suggests that they may represent an untapped resource for such targets, since analogous enzymes share the same activity but possess different tertiary structures, an interesting attribute for drug development.

In previous studies, the existence of functional analogues was observed in several important steps in the metabolism of *T*. *cruzi*, such as the energetic [26] and amino acids pathways [27]. These works show enzymes that are analogous to those found in the human host, listed as possible new therapeutic targets to be studied. Other studies of analogous enzymes have suggested they comprise about 25% of the total enzymatic activity of an organism [28].

In this work, the protein sequences that have been predicted from the *T*. *cruzi *genome sequence were analysed with the objective of improving the annotation of their putative biological functions, and to model their probable three-dimensional structures. We used a high-throughput computational environment that uses comparative modelling techniques for 3D protein structure prediction. In our comparison of *T*. *cruzi *and *Homo sapiens *enzyme sequences, we could identify and model the 3D structure of 356 homologous, 3 analogous and 38 specific *T*. *cruzi *putative enzymes, that can be investigated as potential drug targets for Chagas' disease treatment.

## Results and Discussion

### Analysis of Enzymatic Functions of *Trypanosoma cruzi *and Construction of Three-Dimensional Models

We intended to perform a comparative analysis of 3D structures for *T. cruzi *and human enzymes, in order to detect significant differences that can be exploited and justify these enzymes as potential drug targets. As a starting point, we used the *T. cruzi *CL-Brener database http://tcruzidb.org/tcruzidb/ of predicted proteins, containing 19,607 entries (translated CDS - Coding Sequences). To remove redundant and very similar sequences, an all-against-all BLAST analysis was done and the output was submitted to BioParser [29]. From multiple sequences with more than 95% identity only one member was kept, resulting in a dataset of 12,348 protein sequences.

These were submitted to the MHOLline workflow http://www.mholline.lncc.br to construct 3D protein structure models by comparative modelling. This analysis resulted in 3,286 models, presented in Table 1, that were classified according to the criterion described in Methods section.

**Table 1:** *Trypanosoma cruzi *3D protein models.

Quality	TOTAL
**1. Very High**	50
**2. High**	200
**3. Good**	79
**4. Medium to Good**	835
**5. Medium to Low**	873
**6. Low**	759
**7. Very Low**	490

**TOTAL**	**3,286**

Number of *Trypanosoma cruzi *proteins that could be modeled by comparative modelling using the MHOLline workflow and their respective quality. The quality of models depends on sequence identity and coverage (See the Table 6 in the Methods for detailed description).

### Inference of Functional Annotation of *Trypanosoma cruzi *Predicted Proteins

We previously reported results [26,27] on the inference of function in proteins predicted from the *T. cruzi *CL-Brener genome initiative http://tcruzidb.org/tcruzidb/ using the annotation module in the AnEnΠ pipeline [28]. In addition to the aforementioned analysis, we have added enzymatic functions specified in Swiss-Prot that were absent in the KEGG database, in order to increase the number of enzymatic functions to be analysed. This was done due to the fact that there are enzymatic functions that are not represented in the metabolic pathways described in the KEGG database (*e.g*. prolineracemase - EC 5.1.1.4).

The choice of the cut-off remains a critical point in this procedure and for this reason we investigated different e-values as cut-off (10*e*^-20^, 10*e*^-40 ^and 10*e*^-80^) in the AnEnΠ methodology (Table 2). In order to confer a high degree of reliability to our analysis we adopted the cut-off of 10*e*^-80 ^for the next steps. To establish a good cut-off we should analyse groups of protein families separately and take into account other parameters like coverage, bit-score and identity, but these is not yet available in AnEnΠ. The inferred protein functions of *T. cruzi *were used to find analogy between the parasite sequences and the predicted proteins of *Homo sapiens*.

**Table 2:** Predicted proteins and enzymatic functions of *Trypanosoma cruzi *using different cutoffs and KEGG and Swiss-Prot databases.

Cutoff	**10*e***^**-20**^		**10*e***^**-40**^		**10*e***^**-80**^	
Database	KEGG	Swiss-Prot	KEGG	Swiss-Prot	KEGG	Swiss-Prot
**Predicted Functions**^*a*^	3,625	2,743	2,805	1,924	1,751	762
**Enzymatic Functions**^*b*^	1,027	749	770	523	517	246

^*a*^Total number of predicted proteins with functions inferred by AnEnΠ

^*b*^Total number of distinct enzymatic functions (EC number) from predicted proteins in ^*a*^.

### **Comparison Between ***Homo sapiens ***and ***Trypanosoma cruzi ***Enzymatic Functions**

**Figure 1:**
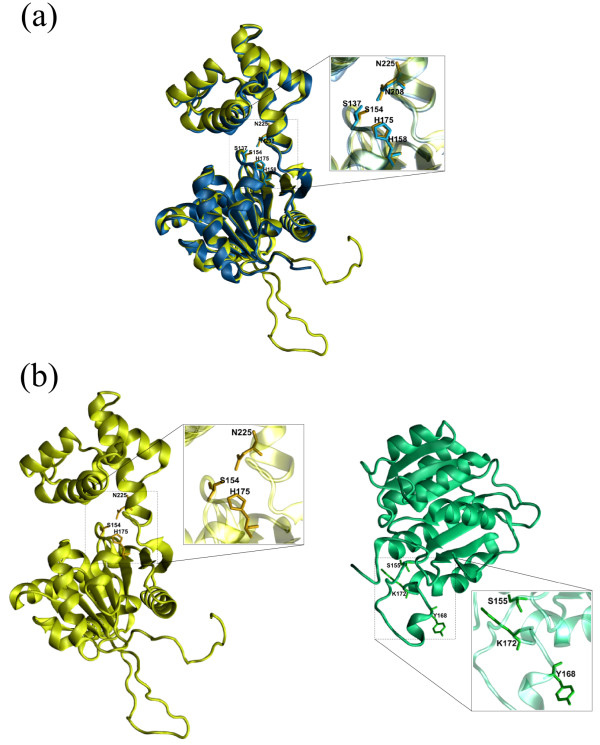
**Structural comparison between a medium to high quality model of 3-Hydroxiacyl-CoA Dehydrogenase from *Trypanosoma cruzi *and one homologous and one analogous structure from the PDB (classified according to the AnEnΠ pipeline)**. 1(a): structural alignment of the *T. cruzi *(Tc00.1047053510105.240) protein model (yellow) and a homologous protein (PDB 1F14) from *Homo sapiens *(blue), detailing its active site residues S137, H158 and N208 according to [30]. The alignment was performed by Swiss-PDB Viewer (v4.0.1) [31]. 1(b): structure of *T. cruzi *(Tc00.1047053510105.240) model (yellow) and the analogous enzyme (PDB 1SO8) from *H. sapiens *(green). The putative active site residues S154, H175 and N225 of *T. cruzi *protein (yellow) are presented in detail, inferred by the alignment in Figure 1(a), and the *H. sapiens *(green) active site (S155, Y168 and K172) from [44]. The images were generated using VMD (Visual Molecular Dynamics - v1.8.6) software [45]

Using AnEnΠ, we analysed and compared the predicted protein sequences from *Homo sapiens *and *Trypanosoma cruzi *to establish possible cases of analogy between these two species. For some enzymatic functions, the sequences of *H. sapiens *and *T. cruzi *were allocated in different clusters, representing probable cases of analogy (see the Methods for more details), while sequences allocated in the same cluster were considered homologous. We expected the 3D structures to be dissimilar in the first case, and probably similar in the latter. This is indeed true in some cases, as exemplified in Figure 1. Also, some sequences are specific to *T. cruzi *and are absent in *H. sapiens*. The results are summarised in Table 3 and were acquired using as final dataset the 478 entries obtained by the combination of both KEGG and Swiss-Prot databases, considering the complete four-digit EC number.

**Table 3:** Comparison between *Homo sapiens *and *Trypanosoma cruzi *functions obtained from KEGG and Swiss-Prot databases.

AnEnΠ Classification	KEGG	Swiss-Prot
**Homologous**^*a*^	356(107)	194 (71)
**Analogous**	28 (5)	8 (6)
**Specific of *T. cruzi***	133 (6)	44 (7)

Numbers in parenthesis represent the number of enzymatic functions (EC number) found among the modelled proteins from *T. cruzi*, using a cut-off of 10*e*^-80^.

^*a*^In some cases, a given protein of the parasite is analogous to a human protein but it also has an homologous counterpart. These cases were included here.

Figure 1 shows examples of comparison between *T. cruzi *and *H. sapiens *protein structures, using the functional classification determined by AnEnΠ Figure 1(a) presents the structural alignment ($RMSD_{C_{\alpha }}$ = 0.65 *Å*) between the *T. cruzi *protein model (yellow), obtained with MHOLline, and the homologous structure (PDB 1F14) of L-3-Hydroxiacyl-CoA Dehydrogenase from *Homo sapiens *(blue). The structure of the active site (S137, H158 and N208) of the human protein, according to [30], is quite similar to the structure of the modelled *T. cruzi *protein. Figure 1(b) shows the same *T. cruzi *model (yellow) and the analogous enzyme (PDB 1SO8) 3-Hydroxiacyl-CoA Dehydrogenase Type II from *H. sapiens *(green). In this figure, the dissimilarity between these two structures is evident. The $RMSD_{C_{\alpha }}$ was calculated using Swiss-PDB Viewer (v4.0.1) program [31].

### Functional Classification of Modelled Enzymes

In the next step, we combined the results presented in Tables 1 and 3, and identified a set of 397 predicted proteins from *Trypanosoma cruzi*, to which an enzymatic function was assigned with the AnEnΠ tool, and for which a structural model was obtained using MHOLline. These functions have 93 distinct EC numbers assigned to them, as showed in Additional file 1, Table S1.

Table 4 summarises the results of the overall analysis in this work. The modelled proteins associated to an EC number were grouped as follows with regard to the comparison between *T. cruzi *and *H. sapiens*: (i) Homologous enzymes; (ii) Analogous enzymes; (iii) Specific of *T. cruzi *and (iv) Undetermined enzymes - enzymes with conflicting clustering depending on the KEGG or Swiss-Prot database used for initial clustering. Moreover, these protein sequences were classified according to IUBMB Nomenclature with regard to the first EC number digit and from 1 to 7 according to the MHOLline model quality proposed in Methods.

**Table 4:** Protein Models: AnEn*π *and enzyme classifications, and model quality.

AnEnΠ	Enzyme Classes	Quality Models							TOTAL
		1	2	3	4	5	6	7	
**1. Homologous**	**Oxidorreductases**	5	16	-	25	8	4	1	**59 **(trypanothione-disulfide reductase)
	**Transferases**	7	17	3	41	12^*b*^	15	9	**104 **(protein kinases, polymerases)
	**Hydrolases**	-	9	5	38	17	14	10	**93 **(trans-sialidase, endopeptidases)
	**Lyases**	-	12	1	1	4	2	-	**20 **(hydratases, endonucleases)
	**Isomerases**	-	1	3	8	1	7	2	**22 **(peptidylprolyl isomerase)
	**Ligases**	-	8	1	14	-	5	5	**33 **(glutathione synthase, ubiquitins)
									
**2. Analogous**	**Oxidorreductases**	-	-	-	1	1	-	-	**2 **(dehydrogenases)
	**Hydrolases**	-	-	-	1	-	-	-	**1 **(phosphatases)
									
**3. Specific of *T. cruzi***	**Oxidorreductases**	1	-	1	-	-	-	-	**2 **(trypanothione-disulfide reductase)
	**Transferases**	-	-	-	2	-	-	-	**2 **(protein kinases, polymerases)
	**Hydrolases**	-	-	2	23	1	4	4	**34 **(cruzipain, leishmanolisin)
									
**4. Undetermined**^*a*^	**Hydrolases**	-	-	-	22	-	-	-	**22 **(leishmanolisin)
	**Lyases**	-	-	-	-	-	-	3	**3 **(hydratases, endonucleases)

**TOTAL**		**13**	**63**	**16**	**176**	**44**	**51**	**34**	**397**

Examples of proteins found in final dataset are presented in parenthesis.

^*a *^Conflicting clustering between results obtained by KEGG and Swiss-Prot databases using AnEnΠ methodology.

^*b *^Two sequences were identified as conflicting annotation between the methodology proposed in this work and GeneDB.

## Discussion and Conclusions

Knowledge of the three-dimensional structures of proteins opens the way to accelerate drug discovery [19]. Theoretical predictions of 3D protein structures and protein folding patterns, even on a genome scale, can provide valuable information to infer possible protein functions and contribute to the identification of potential drug targets [32]. It is believed that evolution tends to conserve functions primarily on the preservation of the 3D structure rather than primary structure. A 3D alignment between structural relatives, even (or mainly) comprising a small number of residues within a protein active site, can be a powerful method to infer function [33].

Using the 19,607 predicted protein sequences from *Trypanosoma cruzi *CL-Brener genome as the initial dataset, we produced a non-redundant dataset comprising 12,348 sequences. Afterwards, these sequences were submitted to the MHOLline workflow and we were able to construct models for 3,286 sequences (26.6% of the total). 1,164 models (35.4%) have a "medium to good" to a "very high" quality (presented in Table 1), being, therefore, suitable for structure-based drug design projects.

It is important to note that there are problems in the processes of genome assembly and annotation, which involve for example the quality of the produced sequences, errors derived from automatic gene prediction, presence of repetitive regions, lack of usage of controlled vocabulary terms (ontology) and propagation of previous annotation errors.

Until now the genome of *T. cruzi *has not been completely assembled, due to the highly repetitive gene content and the heterozygosity of the *T. cruzi *strain at hand. Many predicted proteins have unknown or putative functions which hinder the correct identification of proteins and consequently the elucidation of the parasite's metabolism. To minimise some of these problems, we used the AnEn pipeline to annotate the *T. cruzi *genome and to identify enzymatic functions using KEGG and Swiss-Prot databases (Table 2). From the comparison between *T. cruzi *and *Homo sapiens *enzymatic functions, we identified a set of 397 *T. cruzi *modelled sequences, comprising 93 distinct EC numbers (see Additional file 1, Table S1). Six sequences originally annotated (by GeneDB) as hypothetical proteins could be associated to an enzymatic function by AnEnΠ (more details in Additional file 2, Table S2).

An important result of this work was the identification and construction of 3D protein models for three sequences classified as analogous and 38 classified as specific for *T. cruzi *(listed on Table 5), which are possibly interesting molecular targets for the development of drugs against Chagas' disease. Among the specific enzymes, we identified some proteins that are already being studied as drug targets (*e.g*. cruzipain and trypanothione-disulfide reductase). It is important to note that the quality of some 3D models constructed for these well known drug targets were classified, by MHOLline, from "medium to good" to "very low". It is due to the fact that the MHOLline model quality considers the total query length for coverage calculation, and not only the portion of sequence aligned via BLAST. The way proteins are assembled could influence the calculation of the alignment's coverage since the length of these sequences could differ from those experimentally solved (*e.g*. the presence of pre- and/or pro-domains in the annotated sequence).

**Table 5:** List of modelled sequences classified by AnEnΠ as analogous or specific of *Trypanosoma cruzi*, in relation to *Homo sapiens*.

Categories	Quality Models	**EC**^***a***^	**Description**^***b***^
**A. Analogous**	4	1.3.1.34	2,4-dienoyl-CoA reductase(NADPH) (ID^*c*^: Tc00.1047053509941.100)
	5	1.3.1.34	2,4-dienoyl-CoA reductase(NADPH) (ID^*c*^: Tc00.1047053510303.210)
			
	6	3.1.1.3	Triacylglycerol lipase (ID^*c*^: Tc00.1047053509005.50)
			
**B. Specific of***T. cruzi*	1	1.8.1.12	Trypanothione-disulfide reductase (ID^*c*^: Tc00.1047053503555.30)
	3	1.8.1.12	Trypanothione-disulfide reductase (ID^*c*^: Tc00.1047053504507.5)
			
	4	2.5.1.47	Cysteine synthase (ID^*c*^: Tc00.1047053507165.50, Tc00.1047053507793.20)
			
	3	3.4.22.51	Cruzipain (ID^*c*^: Tc00.1047053508595.50, Tc00.1047053507297.10)
	6	3.4.22.51	Cruzipain (ID^*c*^: Tc00.1047053506529.550, Tc00.1047053507537.20)
	7	3.4.22.51	Cruzipain (ID^*c*^: Tc00.1047053509429.320, Tc00.1047053507603.260, Tc00.1047053507603.270, Tc00.1047053509401.30)
			
	5	3.6.3.6	Proton-exporting ATPase (ID^*c*^: Tc00.1047053506649.20)
	6	3.6.3.6	Proton-exporting ATPase (ID^*c*^: Tc00.1047053505763.19)
			
	4	3.4.24.36	Leishmanolysin (ID^*c*^: Tc00.1047053511211.90, Tc00.1047053510565.150, Tc00.1047053507623.110, Tc00.1047053508699.100, Tc00.1047053508699.90, Tc00.1047053509011.80, Tc00.1047053506587.100, Tc00.1047053509205.100, Tc00.1047053506163.10, Tc00.1047053506163.20, Tc00.1047053508813.40, Tc00.1047053505965.10, Tc00.1047053506257.50, Tc00.1047053510899.10, Tc00.1047053505931.10, Tc00.1047053505931.20, Tc00.1047053511203.10, Tc00.1047053504397.20, Tc00.1047053506921.10, Tc00.1047053508475.30, Tc00.1047053505615.10, Tc00.1047053508825.10, Tc00.1047053510873.20, Tc00.1047053507197.10)

^*a *^EC number determined by AnEnΠ methodology.

^*b *^EC number description obtained from Swiss-Prot database.

^*c *^*Trypanosoma cruzi *identification number according to TcruziDB (version 5.0).

In general, to confirm the potential of these 41 proteins as structure-based drug targets, it is necessary to take into account the importance of metabolic pathways involved in parasite survival, the existence of possible isoforms and alternative metabolic pathways, data about enzymatic assays and the quality of constructed model for further structural analysis, and other information that could help in understanding the physico-chemical properties, catalytic sites and pharmacological inhibitors of these proteins. Of course, one should not discard the 356 sequences classified as homologous proteins in relation to *H. sapiens *glyceraldehyde-3-phosphate dehydrogenase [34], for example, is an important known drug target.

We have further analysed the models for the *T. cruzi *analogous enzymes (presented in Table 5) 2,4-dienoyl-CoA reductase (EC 1.3.1.34) and triacylglycerol lipase (EC 3.1.1.3), which are involved in the metabolism of lipids. The major aspects of lipid metabolism concern fatty acid oxidation to produce energy, and the synthesis of lipids. Knowledge about the oxidation of fatty acids as a source of ATP for trypanosomatids remains scarce. Previous analysis of *T. brucei*, *T. cruzi *and *Leishmania *genomes identified orthologous genes encoding enzymes involved in the *β*-oxidation of fatty acids, and this pathway probably occurs in both glycosomes and mitochondria [35].

**Figure 2:**
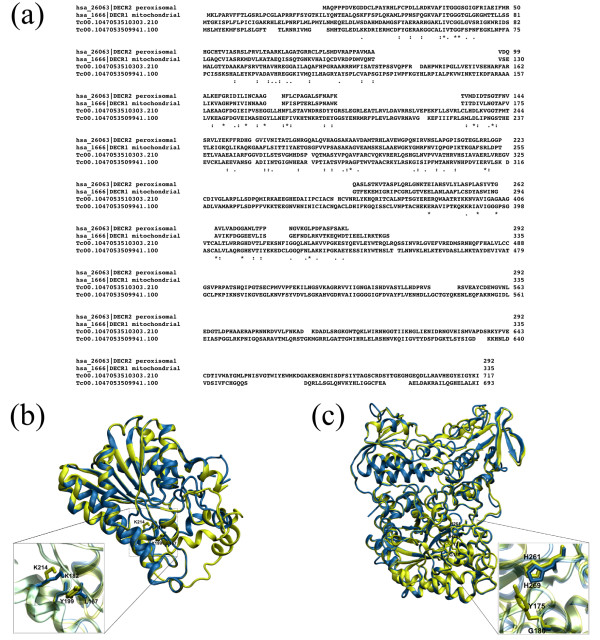
**Structural and sequence comparison between 2,4-dienoyl CoA reductase (DECR) from *Trypanosoma cruzi *and *Homo sapiens*, analogous enzymes**. 2(a): sequence alignment between putative paralogous DECR enzymes from *T. cruzi *and mitochondrial DECR1 and peroxisomal DECR2 enzymes from *H. sapiens*. The alignment was performed using ClustalX (v1.83) [46]. 2(b): structural alignment between the *H. sapiens *DECR1 (reconstructed PDB: 1W6U) (yellow) and DECR2 model (blue), constructed using PDB: 1W6U as template. The active site residues Y199 and K214 of DECR1 (yellow) are presented in detail, according to [47], and L167 and K182 of DECR2 (blue), which were inferred by the structural alignment with DECR1. 2(c): structural alignment between DECR enzymes of *T. cruzi*. The putative active sites constituted by Y175 and H261 of Tc00.1047053509941.100 (yellow) and, G180 and H269 of Tc00.1047053510303.210 (blue) are presented in detail. The active sites of *T. cruzi *DECR were inferred by their structural alignment (not presented) with the DECR protein (PDB: 1PS9) from *Escherichia coli*, used as template. Its active site residues Y166 and H252 are described by [36]. The alignments were performed by Swiss-PDB Viewer (v4.0.1) program [31] and the images were generated using VMD (Visual Molecular Dynamics - v1.8.6) software [45]

The oxidation of polyunsaturated fatty acids requires an auxiliary enzyme (2,4-dienoyl-CoA reductase) that removes the double bonds in the fatty acids. This enzyme (combined with enoyl-CoA isomerase) is essential to allow beta-oxidation and consequently energy production for the parasite [36]. It is possible that this reaction occurs in the opposite direction, generating an unsaturation which could be important in the synthesis of a compound produced in the parasite, whenever the parasite requires it in the composition of unsaturated fatty acids. The sequence and structure alignment between the two isoforms of 2,4-dienoyl-CoA reductase from *T. cruzi *suggest that these proteins are paralogous. Figure 2 presents the difference between the primary and tertiary structures of the paralogous enzymes of *T. cruzi *and the 2,4-dienoyl CoA reductase 1 (DECR1 - mitochondrial) and 2,4-dienoyl CoA reductase 2 (DECR2 - peroxisomal) of *H. sapiens*.

The other analogous enzyme, triacylglycerol lipase, converts triacylglycerol and H_2_O into diacylglycerol and a carboxylate. This reaction is important to glycerolipid metabolism [37] showed that the parasite is able to take up LDL cholesterol (by endocytosis), a molecule that has triglycerides in its composition, justifying the presence of this enzyme in the parasite. Furthermore, the product of this reaction is diacylglycerol, an important molecule for the synthesis of membrane lipids (phospholipids and glycolipids). Taking into account the presented results and the importance of the two enzymatic activities in the oxidation of polyunsaturated fatty acids and glycerolipid metabolism, these analogous enzymes might be an interesting choice for further studies for drug development against Chagas' disease.

The most widely used paradigm in the search of new drug targets is to look for pathogen specific molecules, against which to develop ligands to inactivate target function without a effecting the host [20]. However, data on the frequency and distribution of analogous enzymes suggest that these enzymes should be studied as additional targets since they are expected to share the same enzymatic activity with sufficiently different tertiary structures, a prerequisite for the development of drugs [20].

The results presented in this work corroborate the idea that structural analysis could be an attractive computational methodology for predicting protein functions [38]. The combination of MHOLline workflow with the AnEnΠ pipeline was effective to infer protein function and to detect and construct structural models of proteins in high-throughput analysis. Thus, we were able to identify a list of *T. cruzi *specific or analogous enzymes that can be considered as target candidates suitable to be used in further structure-based drug design projects against Chagas' disease (a complete list of proteins is provided in Additional file 2, Table S2).

## Methods

### Datasets

In this work, we used a dataset composed of 19,607 predicted protein sequences from the *Trypanosoma cruzi *genome (CL-Brener strain). This dataset was obtained from TcruziDB http://tcruzidb.org/common/downloads/release-5.0/Tcruzi/TcruziAnnotatedProtein.fas - version 5.0. AnEnΠ is a tool for identification and annotation of analogous enzymes [28]. We have used the dataset contained in AnEnΠ (Analogous Enzyme Pipeline), which was obtained from the KEGG (Kyoto Encyclopedia of Genes and Genomes) database (from ftp://ftp.genome.ad.jp/pub/kegg/ of December, 2006) [39]. To increase the number of identifiable enzymatic functions by AnEnΠ, we incorporated data from Swiss-Prot [40] (from http://www.expasy.org/sprot/ of May, 2008), resulting in a final dataset composed of 478 four-digit EC numbers. Each *T. cruzi *enzyme function obtained (considering the complete four-digit EC number) was compared with the original genome function annotation list from GeneDB (from http://www.genedb.org/ of October, 2007).

The structures used as templates to provide 3D models of predicted proteins from *T. cruzi *were obtained from the Protein Data Bank (PDB) (44,700 sequences from ftp://ftp.wwpdb.org/pub/pdb/derived_data/ of December, 2006). These models were constructed by comparative modelling method using the workflow MHOLline, as described in the Methods.

### High-Throughput Comparative Modelling

To construct 3D structural models of the predicted proteins from the *T. cruzi *genome we used the MHOLline software http://www.mholline.lncc.br, a biological workflow that combines a specific set of programs for automated protein structure prediction, detection of transmembrane regions, and EC number association. It extracts distinct and valuable structural information about protein sequences even in large-scale genome annotation projects

MHOLline uses the HMMTOP program to identify transmembrane regions. The BLAST algorithm is used for template structure identification by performing searches against the Protein Data Bank [41]. Refinements in the template search - a key step for the model construction - were implemented with the development of a program called BATS (Blast Automatic Targeting for Structures). BATS identifies the sequences where comparative modelling techniques can be applied, by choosing template sequences from the BLAST output file using their scores, expectation values, identity and sequence similarity as criteria. It also consider the number of gaps and the alignment coverage.

BATS also selects the best template for 3D model construction and generates the files for the automated alignment used by the Modeller program [42]. The generated models are evaluated by stereochemical quality using the Procheck program [43]. In summary, for each submitted sequence, MHOLline generates and aggregates structural information, returns a 3D model, a Ramachandran plot and comments about structure quality and enzymatic function.

#### Sequence Filtering and Generation of Distinct Quality Protein Models

To exclude possibly redundant and very similar sequences, an all-against-all BLAST analysis was performed in the dataset composed of all *T. cruzi *translated CDS, using the BLOSUM62 matrix and an e-value ≤ 10*e*^-5 ^as cutoff. The result was automatically filtered by identity (≤ 95%) using the BioParser tool [29]. This non redundant dataset of *T. cruzi *was submitted to the MHOLline workflow to construct the 3D protein models. Sequences were locally aligned by MHOLline (using BLASTP) with protein sequences from PDB using an e-value ≤ 10*e*^-5^. The MHOLline program filtered the new set of aligned sequences with the BATS program and the Filters tool, and it constructed the protein structure models using the Modeller program. Table 6 displays the criteria used for the classification of the obtained models.

**Table 6:** Classification according to the quality of the models built based on BLAST sequence identity and BATS coverage of the template in relation to the target.

Quality	Identity	Coverage
**1. Very High**	≥ 75%	≥ 90%
**2. High**	≥ 50% and < 75%	≥ 90%
**3. Good**	≥ 50%	≥ 70% and < 90%
**4. Medium to Good**	≥ 35% and < 50%	≥ 70%
**5. Medium to Low**	≥ 25% and < 35%	≥ 70%
**6. Low**	≥ 25%	≥ 50% and < 70%
**7. Very Low**	≥ 25%	≥ 30% and < 50%

### *Trypanosoma cruzi ***Protein Function Inference**

AnEnΠ uses the similarity score of BLASTP pairwise comparisons between all proteins included in a previously determined group to assign these proteins to separate clusters for each enzymatic function (EC numbers). Enzymes inside a cluster are considered homologous, while enzymes in different clusters (of the same group/function) are considered analogous.

With the purpose of annotation and identification, users can perform similarity searches by BLASTP. In this case, the database is composed of the sequences belonging to each cluster. In this study, AnEnΠ was used for the identification of predicted proteins of *Trypanosoma cruzi *using different e-values as cutoff (10*e*^-20^, 10*e*^-40 ^and 10*e*^-80^).

## Authors' contributions

PVSZC and ACRG performed all computational analysis, and drafted the manuscript. TDO performed some computational analysis related to analogies. ABM, LED and WMD planned and supervised the study. All authors read and approved the final manuscript.

## Supplementary Material

Additional file 1 Table S1 - Enzyme Commission Numbers (EC) associated to modelled *Trypanosoma cruzi *proteins. Click here for file

Additional file 2 Table S2 - Complete list of homologous, analogous and specific 3D protein models of *Trypanosoma cruzi *versus *Homo sapiens*. Click here for file
