# Supplementary material for: Structural modelling and comparative analysis of homologous, analogous and specific proteins from Trypanosoma cruzi versus Homo sapiens: putative drug targets for chagas' disease treatment
Source: BMC Genomics. 2010 Oct 29;11:610. doi: 10.1186/1471-2164-11-610 (PMC3091751; doi:10.1186/1471-2164-11-610)
Supplement: Additional file 1 — Table S1 - Enzyme Commission Numbers (EC) associated to modelled Trypanosoma cruzi proteins. [file 1471-2164-11-610-S1.PDF]

Table S1 - Enzyme Commission Numbers (EC) associated to modelled *Trypanosoma cruzi* proteins.

EC[a]	Description	Total ID[b]	EC[a]	Description	Total ID[b]
1.1.1.205	IMP dehydrogenase	2	3.1.1.3	Triacylglycerol lipase	1
1.1.1.271	GDP-L-fucose synthase	1	3.1.1.29	Aminoacyl-tRNA hydrolase	1
1.1.1.35	3-hydroxyacyl-CoA dehydrogenase	2	3.1.2.6	Hydroxyacylglutathione hydrolase	2
1.1.1.37	Malate dehydrogenase	6	3.1.2.15	Ubiquitin thiolesterase	9
1.1.1.40	Malate dehydrogenase (oxaloacetate-decarboxylating)(NADP(+))	4	3.1.3.11	Fructose-bisphosphatase	2
1.1.1.42	Isocitrate dehydrogenase (NADP(+))	2	3.1.3.16	Phosphoprotein phosphatase	23
1.1.1.44	Phosphogluconate dehydrogenase (decarboxylating)	1	3.1.3.25	Inositol-phosphate phosphatase	2
1.11.1.9	Glutathione peroxidase	1	3.1.3.48	Protein-tyrosine-phosphatase	11
1.11.1.15	Peroxiredoxin	1	3.2.1.18	Exo-alpha-sialidase	1
1.15.1.1	Superoxide dismutase	5	3.4.11.1	Leucyl aminopeptidase	2
1.2.1.3	Aldehyde dehydrogenase (NAD(+))	1	3.4.11.18	Methionyl aminopeptidase	4
1.2.1.5	Aldehyde dehydrogenase (NAD(P)(+))	2	3.4.21.26	Prolyl oligopeptidase	1
1.2.1.12	Glyceraldehyde-3-phosphate dehydrogenase (phosphorylating)	10	3.4.22.51	Cruzipain	8
1.2.4.1	Pyruvate dehydrogenase (acetyl-transferring)	4	3.4.24.16	Neurolysin	2
1.2.4.4	3-methyl-2-oxobutanoate dehydrogenase (2-methylpropanoyl-transferring)	2	3.4.24.36	Leishmanolysin	56
1.3.1.34	2,4-dienoyl-CoA reductase(NADPH)	2	3.4.24.56	Insulysin	2
1.3.99.3	Acyl-CoA dehydrogenase	2	3.4.25.1	Proteasome endopeptidase complex	4
1.6.2.2	Cytochrome-b5 reductase	7	3.5.1.88	Peptide deformylase	3
1.6.2.4	NADPH–hemoprotein reductase	2	3.5.4.6	AMP deaminase	9
1.8.1.4	Dihydrolipoyl dehydrogenase	4	3.6.1.1	Inorganic diphosphatase	2
1.8.1.12	Trypanothione-disulfide reductase	2	3.6.3.14	H(+)-transporting two-sector ATPase	1
2.3.1.7	Carnitine O-acetyltransferase	1	3.6.3.6	Proton-exporting ATPase	2
2.3.1.12	Dihydrolipoyllysine-residue acetyltransferase	1	3.6.4.6	Vesicle-fusing ATPase	2
2.3.1.29	Glycine C-acetyltransferase	4	4.1.1.35	UDP-glucuronate decarboxylase	2
2.3.1.61	Dihydrolipoyllysine-residue succinyltransferase	2	4.1.1.50	Adenosylmethionine decarboxylase	1
2.3.2.5	Glutaminyl-peptide cyclotransferase	2	4.2.1.17	Enoyl-CoA hydratase	2
2.4.2.29	tRNA-guanine transglycosylase	2	4.2.1.22	Cystathionine beta-synthase	10
2.4.2.30	NAD(+)ADP-ribosyl transferase	2	4.2.1.47	GDP-mannose 4,6-dehydratase	1
2.5.1.46	Deoxyhypusine synthase	1	4.2.99.18	DNA-(apurinic or apyrimidinic site) lyase	4
2.5.1.47	Cysteine synthase	2	4.6.1.1	Adenylate cyclase	3
2.5.1.60	Protein geranylgeranyltransferase typeII	1	5.1.1.4	Proline racemase	1
2.6.1.1	Aspartate transaminase	3	5.1.3.1	Ribulose-phosphate 3-epimerase	2
2.6.1.5	Tyrosine transaminase	7	5.2.1.8	Peptidylprolyl isomerase	12
2.7.1.24	Dephospho-CoA kinase	1	5.3.3.8	Dodecenoyl-CoA isomerase	2
2.7.1.30	Glycerol kinase	1	5.3.4.1	Protein disulfide-isomerase	3
2.7.1.105	6-phosphofructo-2-kinase	2	5.99.1.3	DNA topoisomerase (ATP-hydrolyzing)	2
2.7.2.3	Phosphoglycerate kinase	6	6.1.1.2	Tryptophan–tRNA ligase	5
2.7.4.3	Adenylate kinase	11	6.1.1.6	Lysine–tRNA ligase	3
2.7.4.6	Nucleoside-diphosphate kinase	1	6.1.1.7	Alanine–tRNA ligase	2
2.7.4.8	Guanylate kinase	1	6.1.1.9	Valine–tRNA ligase	2
2.7.6.1	Ribose-phosphate diphosphokinase	3	6.1.1.12	Aspartate–tRNA ligase	3
2.7.7.6	DNA-directed RNA polymerase	4	6.1.1.18	Glutamine–tRNA ligase	2
2.7.7.7	DNA-directed DNA polymerase	9	6.1.1.22	Asparagine–tRNA ligase	1
2.7.11.1	Non-specific serine/threonine protein kinase	23	6.2.1.1	Acetate–CoA ligase	2
2.7.11.22	Cyclin-dependent kinase	7	6.3.2.19	Ubiquitin–protein ligase	13
2.7.11.24	Mitogen-activated protein kinase	5			
2.7.11.26	[Tau protein] kinase	1			
2.7.12.1	Dual-specificity kinase	3			

[a] EC number determined by AnEnΠ methodology.
[b] Amount of *Trypanosoma cruzi* identification number according to TcruziDB (version 5.0).
