# Supplementary material for: Structural modelling and comparative analysis of homologous, analogous and specific proteins from Trypanosoma cruzi versus Homo sapiens: putative drug targets for chagas' disease treatment
Source: BMC Genomics. 2010 Oct 29;11:610. doi: 10.1186/1471-2164-11-610 (PMC3091751; doi:10.1186/1471-2164-11-610)
Supplement: Additional file 2 — Table S2 - Complete list of homologous, analogous and specific 3D protein models of Trypanosoma cruzi versus Homo sapiens. [file 1471-2164-11-610-S2.PDF]

Categories		MHOLline Quality	Query Name
Matching Annotation	Homologous	1	Trypanosoma_cruzi|AAHK01000013|Tc00.1047053510187.30|Annotation|GenBank|(proteincoding)(416letters)
Matching Annotation	Homologous	1	Trypanosoma_cruzi|AAHK01000013|Tc00.1047053510187.40|Annotation|GenBank|(proteincoding)(416letters)
Matching Annotation	Homologous	1	Trypanosoma_cruzi|AAHK01000013|Tc00.1047053510187.50|Annotation|GenBank|(proteincoding)(416letters)
Matching Annotation	Homologous	1	Trypanosoma_cruzi|AAHK01000292|Tc00.1047053505999.90|Annotation|GenBank|(proteincoding)(419letters)
Matching Annotation	Homologous	1	Trypanosoma_cruzi|AAHK01000475|Tc00.1047053509065.60|Annotation|GenBank|(proteincoding)(359letters)
Matching Annotation	Homologous	1	Trypanosoma_cruzi|AAHK01000475|Tc00.1047053509065.70|Annotation|GenBank|(proteincoding)(359letters)
Matching Annotation	Homologous	1	Trypanosoma_cruzi|AAHK01000650|Tc00.1047053506943.50|Annotation|GenBank|(proteincoding)(359letters)
Matching Annotation	Homologous	1	Trypanosoma_cruzi|AAHK01000650|Tc00.1047053506943.60|Annotation|GenBank|(proteincoding)(359letters)
Matching Annotation	Homologous	1	Trypanosoma_cruzi|AAHK01001066|Tc00.1047053511461.20|Annotation|GenBank|(proteincoding)(416letters)
Matching Annotation	Homologous	1	Trypanosoma_cruzi|AAHK01001252|Tc00.1047053511419.40|Annotation|GenBank|(proteincoding)(419letters)
Matching Annotation	Homologous	1	Trypanosoma_cruzi|AAHK01001273|Tc00.1047053510663.70|Annotation|GenBank|(proteincoding)(477letters)
Matching Annotation	Homologous	1	Trypanosoma_cruzi|AAHK01001742|Tc00.1047053510795.10|Annotation|GenBank|(proteincoding)(416letters)
Matching Annotation	Homologous	2	Trypanosoma_cruzi|AAHK01000001|Tc00.1047053508153.130|Annotation|GenBank|(proteincoding)(267letters)
Matching Annotation	Homologous	2	Trypanosoma_cruzi|AAHK01000011|Tc00.1047053506925.300|Annotation|GenBank|(proteincoding)(177letters)
Matching Annotation	Homologous	2	Trypanosoma_cruzi|AAHK01000011|Tc00.1047053506925.319|Annotation|GenBank|(proteincoding)(413letters)
Matching Annotation	Homologous	2	Trypanosoma_cruzi|AAHK01000013|Tc00.1047053510187.60|Annotation|GenBank|(proteincoding)(338letters)
Matching Annotation	Homologous	2	Trypanosoma_cruzi|AAHK01000029|Tc00.1047053508175.360|Annotation|GenBank|(proteincoding)(401letters)
Matching Annotation	Homologous	2	Trypanosoma_cruzi|AAHK01000043|Tc00.1047053507993.80|Annotation|GenBank|(proteincoding)(353letters)
Matching Annotation	Homologous	2	Trypanosoma_cruzi|AAHK01000057|Tc00.1047053509965.380|Annotation|GenBank|(proteincoding)(325letters)
Matching Annotation	Homologous	2	Trypanosoma_cruzi|AAHK01000073|Tc00.1047053510105.230|Annotation|GenBank|(proteincoding)(338letters)
Matching Annotation	Homologous	2	Trypanosoma_cruzi|AAHK01000085|Tc00.1047053510303.290|Annotation|GenBank|(proteincoding)(401letters)
Matching Annotation	Homologous	2	Trypanosoma_cruzi|AAHK01000091|Tc00.1047053504427.110|Annotation|GenBank|(proteincoding)(677letters)
Matching Annotation	Homologous	2	Trypanosoma_cruzi|AAHK01000104|Tc00.1047053510421.320|Annotation|GenBank|(proteincoding)(347letters)
Matching Annotation	Homologous	2	Trypanosoma_cruzi|AAHK01000124|Tc00.1047053507089.270|Annotation|GenBank|(proteincoding)(477letters)
Matching Annotation	Homologous	2	Trypanosoma_cruzi|AAHK01000172|Tc00.1047053511903.30|Annotation|GenBank|(proteincoding)(156letters)
Matching Annotation	Homologous	2	Trypanosoma_cruzi|AAHK01000179|Tc00.1047053509233.180|Annotation|GenBank|(proteincoding)(518letters)
Matching Annotation	Homologous	2	Trypanosoma_cruzi|AAHK01000183|Tc00.1047053509695.190|Annotation|GenBank|(proteincoding)(301letters)
Matching Annotation	Homologous	2	Trypanosoma_cruzi|AAHK01000243|Tc00.1047053509331.30|Annotation|GenBank|(proteincoding)(677letters)
Matching Annotation	Homologous	2	Trypanosoma_cruzi|AAHK01000307|Tc00.1047053508541.220|Annotation|GenBank|(proteincoding)(312letters)
Matching Annotation	Homologous	2	Trypanosoma_cruzi|AAHK01000307|Tc00.1047053508541.230|Annotation|GenBank|(proteincoding)(330letters)
Matching Annotation	Homologous	2	Trypanosoma_cruzi|AAHK01000307|Tc00.1047053508541.240|Annotation|GenBank|(proteincoding)(330letters)
Matching Annotation	Homologous	2	Trypanosoma_cruzi|AAHK01000328|Tc00.1047053508815.110|Annotation|GenBank|(proteincoding)(303letters)
Matching Annotation	Homologous	2	Trypanosoma_cruzi|AAHK01000330|Tc00.1047053509717.80|Annotation|GenBank|(proteincoding)(338letters)
Matching Annotation	Homologous	2	Trypanosoma_cruzi|AAHK01000336|Tc00.1047053511301.110|Annotation|GenBank|(proteincoding)(512letters)
Matching Annotation	Homologous	2	Trypanosoma_cruzi|AAHK01000346|Tc00.1047053509153.120|Annotation|GenBank|(proteincoding)(401letters)
Matching Annotation	Homologous	2	Trypanosoma_cruzi|AAHK01000409|Tc00.1047053510609.70|Annotation|GenBank|(proteincoding)(330letters)
Matching Annotation	Homologous	2	Trypanosoma_cruzi|AAHK01000457|Tc00.1047053511019.90|Annotation|GenBank|(proteincoding)(195letters)
Matching Annotation	Homologous	2	Trypanosoma_cruzi|AAHK01000459|Tc00.1047053511071.140|Annotation|GenBank|(proteincoding)(404letters)
Matching Annotation	Homologous	2	Trypanosoma_cruzi|AAHK01000459|Tc00.1047053511071.70|Annotation|GenBank|(proteincoding)(156letters)
Matching Annotation	Homologous	2	Trypanosoma_cruzi|AAHK01000516|Tc00.1047053506457.10|Annotation|GenBank|(proteincoding)(475letters)
Matching Annotation	Homologous	2	Trypanosoma_cruzi|AAHK01000517|Tc00.1047053506201.30|Annotation|GenBank|(proteincoding)(330letters)
Matching Annotation	Homologous	2	Trypanosoma_cruzi|AAHK01000517|Tc00.1047053506201.70|Annotation|GenBank|(proteincoding)(297letters)
Matching Annotation	Homologous	2	Trypanosoma_cruzi|AAHK01000517|Tc00.1047053506201.80|Annotation|GenBank|(proteincoding)(294letters)
Matching Annotation	Homologous	2	Trypanosoma_cruzi|AAHK01000518|Tc00.1047053511575.60|Annotation|GenBank|(proteincoding)(433letters)
Matching Annotation	Homologous	2	Trypanosoma_cruzi|AAHK01000520|Tc00.1047053510761.60|Annotation|GenBank|(proteincoding)(345letters)
Matching Annotation	Homologous	2	Trypanosoma_cruzi|AAHK01000521|Tc00.1047053508241.140|Annotation|GenBank|(proteincoding)(384letters)
Matching Annotation	Homologous	2	Trypanosoma_cruzi|AAHK01000545|Tc00.1047053511179.160|Annotation|GenBank|(proteincoding)(148letters)
Matching Annotation	Homologous	2	Trypanosoma_cruzi|AAHK01000552|Tc00.1047053508177.110|Annotation|GenBank|(proteincoding)(384letters)
Matching Annotation	Homologous	2	Trypanosoma_cruzi|AAHK01000552|Tc00.1047053508177.120|Annotation|GenBank|(proteincoding)(384letters)

Annotation (by GeneDB)	PDB ID	PDB CHAIN	ECN (by AnEnPi)	ECN Description (by Swiss-Prot)
tyrosineaminotransferase	1BW0	B	2.6.1.5	Tyrosinetransaminase
tyrosineaminotransferase	1BW0	B	2.6.1.5	Tyrosinetransaminase
tyrosineaminotransferase	1BW0	B	2.6.1.5	Tyrosinetransaminase
phosphoglyceratekinase,putative	16PK	1	2.7.2.3	Phosphoglyceratekinase
glyceraldehyde3-phosphatedehydrogenase,putative	1QXS	B	1.2.1.12	Glyceraldehyde-3-phosphatedehydrogenase(phosphorylating)
glyceraldehyde3-phosphatedehydrogenase,putative	1QXS	B	1.2.1.12	Glyceraldehyde-3-phosphatedehydrogenase(phosphorylating)
glyceraldehyde3-phosphatedehydrogenase,putative	1QXS	B	1.2.1.12	Glyceraldehyde-3-phosphatedehydrogenase(phosphorylating)
glyceraldehyde3-phosphatedehydrogenase,putative	1QXS	B	1.2.1.12	Glyceraldehyde-3-phosphatedehydrogenase(phosphorylating)
tyrosineaminotransferase	1BW0	B	2.6.1.5	Tyrosinetransaminase
phosphoglyceratekinase,putative	16PK	1	2.7.2.3	Phosphoglyceratekinase
6-phosphogluconatedehydrogenase,decarboxylating,putative	1PGJ	B	1.1.1.44	Phosphogluconatedehydrogenase(decarboxylating)
tyrosineaminotransferase	1BW0	B	2.6.1.5	Tyrosinetransaminase
enoyl-CoAhydratase,mitochondrialprecursor,putative	1ZNQ	F	4.2.1.17	Enoyl-CoAhydratase
cyclophilinA,PPIase,rotamase,peptidyl-prolylcis-transisomerase	1ZNQ	B	5.2.1.8	Peptidylprolylisomerase
isocitratedehydrogenase,putative	1LWD	A	1.1.1.42	Isocitratedehydrogenase(NADP(+))
glyceraldehyde3-phosphatedehydrogenase,cytosolic,putative	1ZNQ	O	1.2.1.12	Glyceraldehyde-3-phosphatedehydrogenase(phosphorylating)
cystathioninebeta-synthase,cysteinesynthase,serinesulfhydrylase	1ZNQ	B	4.2.1.22	Cystathioninebeta-synthase
proteinkinase,putative,glycogensynthasekinase,putative	1ZNQ	A	2.7.11.26	[Tauprotein]kinase
dTDP-glucose4,6-dehydratase,putative	1ZNQ	A	4.1.1.35	UDP-glucuronatedecarboxylase
glyceraldehyde3-phosphatedehydrogenase,cytosolic,putative	1ZNQ	O	1.2.1.12	Glyceraldehyde-3-phosphatedehydrogenase(phosphorylating)
acyl-CoAdehydrogenase,putative	1ZNQ	D	1.3.99.3	Acyl-CoAdehydrogenase
acetyl-CoAsynthetase,putative	1ZNQ	B	6.2.1.1	Acetate--CoAligase
pyruvatedehydrogenaseE1betasubunit,putative	1ZNQ	D	1.2.4.1	Pyruvatedehydrogenase(acetyl-transferring)
dihydrolipoyldehydrogenase,putative	1ZNQ	A	1.8.1.4	Dihydrolipoyldehydrogenase
ubiquitin-conjugatingenzymeE2,putative	1ZNQ	1	6.3.2.19	Ubiquitin--proteinligase
ATPasebetasubunit,putative	1ZNQ	D	3.6.3.14	H(+)-transportingtwo-sectorATPase
celldivisionproteinkinase2,putative	1ZNQ	A	2.7.11.22	Cyclin-dependentkinase
acetyl-CoAsynthetase,putative	1ZNQ	B	6.2.1.1	Acetate--CoAligase
caseinkinase,putative	1ZNQ	B	2.7.11.1	Non-specificserine/threonineproteinkinase
caseinkinase,putative	1ZNQ	A	2.7.11.1	Non-specificserine/threonineproteinkinase
caseinkinase,putative	1ZNQ	A	2.7.11.1	Non-specificserine/threonineproteinkinase
serine/threonineproteinphosphatasecatalyticsubunit,putative	1ZNQ	A	3.1.3.16	Phosphoproteinphosphatase
glyceraldehyde3-phosphatedehydrogenase,cytosolic,putative	1ZNQ	O	1.2.1.12	Glyceraldehyde-3-phosphatedehydrogenase(phosphorylating)
inosine-5'-monophosphatedehydrogenase,putative	1NFB	B	1.1.1.205	IMPdehydrogenase
acyl-CoAdehydrogenase,putative	1ZNQ	D	1.3.99.3	Acyl-CoAdehydrogenase
proteinkinase,putative,cdc2-relatedkinase,putative	1ZNQ	A	2.7.11.22	Cyclin-dependentkinase
ironsuperoxidedismutase,putative	1DT0	C	1.15.1.1	Superoxidedismutase
2-amino-3-ketobutyratecoenzymeAligase,putative	1ZNQ	B	2.3.1.29	GlycineC-acetyltransferase
ubiquitin-conjugatingenzymeE2,putative	1ZNQ	1	6.3.2.19	Ubiquitin--proteinligase
methionineaminopeptidase2,putative,metallo-peptidase,clanMG,familyM24,putative	1ZNQ	1	3.4.11.18	Methionylaminopeptidase
serine/threonine-proteinphosphatasePP1beta	1ZNQ	A	3.1.3.16	Phosphoproteinphosphatase
serine/threonine-proteinphosphatasePP1alpha	1ZNQ	A	3.1.3.16	Phosphoproteinphosphatase
serine/threonine-proteinphosphatasePP1,putative	1ZNQ	A	3.1.3.16	Phosphoproteinphosphatase
isocitratedehydrogenase[NADP],mitochondrialprecursor,putative	1T0L	D	1.1.1.42	Isocitratedehydrogenase(NADP(+))
caseinkinaseII,putative	1ZNQ	A	2.7.11.1	Non-specificserine/threonineproteinkinase
cystathioninebeta-synthase,putative,cysteinesynthase,serinesulfhydrylase	1ZNQ	F	4.2.1.22	Cystathioninebeta-synthase
ubiquitin-conjugatingenzymeE2,putative	1ZNQ	A	6.3.2.19	Ubiquitin--proteinligase
cystathioninebeta-synthase,cysteinesynthase,serinesulfhydrylase	1ZNQ	F	4.2.1.22	Cystathioninebeta-synthase
cystathioninebeta-synthase,cysteinesynthase,serinesulfhydrylase	1ZNQ	F	4.2.1.22	Cystathioninebeta-synthase

Matching Annotation	Homologous	2	Trypanosoma_cruzi|AAHK01000594|Tc00.1047053508303.4|Annotation|GenBank|(proteincoding)(148letters)
Matching Annotation	Homologous	2	Trypanosoma_cruzi|AAHK01000630|Tc00.1047053511025.110|Annotation|GenBank|(proteincoding)(477letters)
Matching Annotation	Homologous	2	Trypanosoma_cruzi|AAHK01000709|Tc00.1047053510091.80|Annotation|GenBank|(proteincoding)(347letters)
Matching Annotation	Homologous	2	Trypanosoma_cruzi|AAHK01001047|Tc00.1047053507883.109|Annotation|GenBank|(proteincoding)(301letters)
Matching Annotation	Homologous	2	Trypanosoma_cruzi|AAHK01001129|Tc00.1047053507757.50|Annotation|GenBank|(proteincoding)(351letters)
Matching Annotation	Homologous	2	Trypanosoma_cruzi|AAHK01001422|Tc00.1047053510381.10|Annotation|GenBank|(proteincoding)(427letters)
Matching Annotation	Homologous	2	Trypanosoma_cruzi|AAHK01001513|Tc00.1047053506905.50|Annotation|GenBank|(proteincoding)(384letters)
Matching Annotation	Homologous	2	Trypanosoma_cruzi|AAHK01001554|Tc00.1047053503617.10|Annotation|GenBank|(proteincoding)(330letters)
Matching Annotation	Homologous	2	Trypanosoma_cruzi|AAHK01001562|Tc00.1047053509499.14|Annotation|GenBank|(proteincoding)(226letters)
Matching Annotation	Homologous	2	Trypanosoma_cruzi|AAHK01001613|Tc00.1047053506583.40|Annotation|GenBank|(proteincoding)(311letters)
Matching Annotation	Homologous	2	Trypanosoma_cruzi|AAHK01001732|Tc00.1047053509051.30|Annotation|GenBank|(proteincoding)(325letters)
Matching Annotation	Homologous	2	Trypanosoma_cruzi|AAHK01001788|Tc00.1047053509633.50|Annotation|GenBank|(proteincoding)(294letters)
Matching Annotation	Homologous	2	Trypanosoma_cruzi|AAHK01001788|Tc00.1047053509633.60|Annotation|GenBank|(proteincoding)(294letters)
Matching Annotation	Homologous	2	Trypanosoma_cruzi|AAHK01001857|Tc00.1047053507211.40|Annotation|GenBank|(proteincoding)(512letters)
Matching Annotation	Homologous	2	Trypanosoma_cruzi|AAHK01001949|Tc00.1047053510647.30|Annotation|GenBank|(proteincoding)(389letters)
Matching Annotation	Homologous	2	Trypanosoma_cruzi|AAHK01001969|Tc00.1047053508137.30|Annotation|GenBank|(proteincoding)(151letters)
Matching Annotation	Homologous	2	Trypanosoma_cruzi|AAHK01002041|Tc00.1047053511899.40|Annotation|GenBank|(proteincoding)(404letters)
Matching Annotation	Homologous	2	Trypanosoma_cruzi|AAHK01002101|Tc00.1047053507541.30|Annotation|GenBank|(proteincoding)(312letters)
Matching Annotation	Homologous	2	Trypanosoma_cruzi|AAHK01002214|Tc00.1047053511691.20|Annotation|GenBank|(proteincoding)(427letters)
Matching Annotation	Homologous	2	Trypanosoma_cruzi|AAHK01002273|Tc00.1047053504181.40|Annotation|GenBank|(proteincoding)(301letters)
Matching Annotation	Homologous	2	Trypanosoma_cruzi|AAHK01002469|Tc00.1047053507305.20|Annotation|GenBank|(proteincoding)(330letters)
Matching Annotation	Homologous	2	Trypanosoma_cruzi|AAHK01002469|Tc00.1047053507305.30|Annotation|GenBank|(proteincoding)(330letters)
Matching Annotation	Homologous	2	Trypanosoma_cruzi|AAHK01002637|Tc00.1047053506677.20|Annotation|GenBank|(proteincoding)(311letters)
Matching Annotation	Homologous	2	Trypanosoma_cruzi|AAHK01002724|Tc00.1047053510027.10|Annotation|GenBank|(proteincoding)(354letters)
Matching Annotation	Homologous	2	Trypanosoma_cruzi|AAHK01003067|Tc00.1047053504929.10|Annotation|GenBank|(proteincoding)(330letters)
Matching Annotation	Homologous	2	Trypanosoma_cruzi|AAHK01005817|Tc00.1047053508185.10|Annotation|GenBank|(proteincoding)(267letters)
Matching Annotation	Homologous	3	Trypanosoma_cruzi|AAHK01000215|Tc00.1047053507031.120|Annotation|GenBank|(proteincoding)(392letters)
Matching Annotation	Homologous	3	Trypanosoma_cruzi|AAHK01000234|Tc00.1047053509693.50|Annotation|GenBank|(proteincoding)(476letters)
Matching Annotation	Homologous	3	Trypanosoma_cruzi|AAHK01000292|Tc00.1047053505999.100|Annotation|GenBank|(proteincoding)(503letters)
Matching Annotation	Homologous	3	Trypanosoma_cruzi|AAHK01000307|Tc00.1047053508541.225|Annotation|GenBank|(proteincoding)(283letters)
Matching Annotation	Homologous	3	Trypanosoma_cruzi|AAHK01000747|Tc00.1047053506795.80|Annotation|GenBank|(proteincoding)(354letters)
Matching Annotation	Homologous	3	Trypanosoma_cruzi|AAHK01000794|Tc00.1047053508897.110|Annotation|GenBank|(proteincoding)(196letters)
Matching Annotation	Homologous	3	Trypanosoma_cruzi|AAHK01000858|Tc00.1047053510755.138|Annotation|GenBank|(proteincoding)(445letters)
Matching Annotation	Homologous	3	Trypanosoma_cruzi|AAHK01001101|Tc00.1047053508413.40|Annotation|GenBank|(proteincoding)(470letters)
Matching Annotation	Homologous	3	Trypanosoma_cruzi|AAHK01001185|Tc00.1047053504111.20|Annotation|GenBank|(proteincoding)(392letters)
Matching Annotation	Homologous	3	Trypanosoma_cruzi|AAHK01001252|Tc00.1047053511419.50|Annotation|GenBank|(proteincoding)(588letters)
Matching Annotation	Homologous	3	Trypanosoma_cruzi|AAHK01001256|Tc00.1047053509149.9|Annotation|GenBank|(proteincoding)(303letters)
Matching Annotation	Homologous	3	Trypanosoma_cruzi|AAHK01001797|Tc00.1047053510259.50|Annotation|GenBank|(proteincoding)(196letters)
Matching Annotation	Homologous	3	Trypanosoma_cruzi|AAHK01003781|Tc00.1047053453445.20|Annotation|GenBank|(proteincoding)(184letters)
Matching Annotation	Homologous	4	Trypanosoma_cruzi|AAHK01000005|Tc00.1047053511277.630|Annotation|GenBank|(proteincoding)(472letters)
Matching Annotation	Homologous	4	Trypanosoma_cruzi|AAHK01000012|Tc00.1047053506885.400|Annotation|GenBank|(proteincoding)(354letters)
Matching Annotation	Homologous	4	Trypanosoma_cruzi|AAHK01000013|Tc00.1047053510187.20|Annotation|GenBank|(proteincoding)(417letters)
Matching Annotation	Homologous	4	Trypanosoma_cruzi|AAHK01000013|Tc00.1047053510187.234|Annotation|GenBank|(proteincoding)(325letters)
Matching Annotation	Homologous	4	Trypanosoma_cruzi|AAHK01000013|Tc00.1047053510187.500|Annotation|GenBank|(proteincoding)(392letters)
Matching Annotation	Homologous	4	Trypanosoma_cruzi|AAHK01000013|Tc00.1047053510187.70|Annotation|GenBank|(proteincoding)(412letters)
Matching Annotation	Homologous	4	Trypanosoma_cruzi|AAHK01000025|Tc00.1047053508355.380|Annotation|GenBank|(proteincoding)(557letters)
Matching Annotation	Homologous	4	Trypanosoma_cruzi|AAHK01000027|Tc00.1047053508741.320|Annotation|GenBank|(proteincoding)(405letters)
Matching Annotation	Homologous	4	Trypanosoma_cruzi|AAHK01000028|Tc00.1047053506435.300|Annotation|GenBank|(proteincoding)(225letters)
Matching Annotation	Homologous	4	Trypanosoma_cruzi|AAHK01000043|Tc00.1047053507993.190|Annotation|GenBank|(proteincoding)(472letters)
Matching Annotation	Homologous	4	Trypanosoma_cruzi|AAHK01000044|Tc00.1047053506855.180|Annotation|GenBank|(proteincoding)(260letters)
Matching Annotation	Homologous	4	Trypanosoma_cruzi|AAHK01000054|Tc00.1047053506247.230|Annotation|GenBank|(proteincoding)(697letters)

ubiquitin-conjugatingenzymeE2,putative	1ZNQ	A	6.3.2.19	Ubiquitin--proteinligase
dihydrolipoyldehydrogenase,putative	1ZNQ	A	1.8.1.4	Dihydrolipoyldehydrogenase
pyruvatedehydrogenaseE1betasubunit,putative	1ZNQ	D	1.2.4.1	Pyruvatedehydrogenase(acetyl-transferring)
mitochondrialmalatedehydrogenase,putative	1SMK	H	1.1.1.37	Malatedehydrogenase
serine/threonine-proteinphosphatasePP1,putative	1ZNQ	A	3.1.3.16	Phosphoproteinphosphatase
cystathioninebeta-synthase,putative	1ZNQ	B	4.2.1.22	Cystathioninebeta-synthase
cystathioninebeta-synthase,cysteinesynthase,serinesulfhydrylase	1ZNQ	F	4.2.1.22	Cystathioninebeta-synthase
proteinkinase,putative,cdc2-relatedkinase,putative	1ZNQ	A	2.7.11.22	Cyclin-dependentkinase
tryparedoxinperoxidase,putative	1ZYE	L	1.11.1.15	Peroxiredoxin
celldivisionrelatedproteinkinase2,putative	1ZNQ	A	2.7.11.22	Cyclin-dependentkinase
dTDP-glucose4,6-dehydratase,putative	1ZNQ	A	4.1.1.35	UDP-glucuronatedecarboxylase
serine/threonine-proteinphosphatasePP1,putative	1ZNQ	A	3.1.3.16	Phosphoproteinphosphatase
serine/threonine-proteinphosphatasePP1,putative	1ZNQ	A	3.1.3.16	Phosphoproteinphosphatase
inosine-5'-monophosphatedehydrogenase,putative	1NFB	B	1.1.1.205	IMPdehydrogenase
tryptophanyl-tRNAsynthetase,putative	1ZNQ	B	6.1.1.2	Tryptophan--tRNAligase
ubiquitin-conjugatingenzymeE2,putative	1ZNQ	B	6.3.2.19	Ubiquitin--proteinligase
2-amino-3-ketobutyratecoenzymeAligase,putative	1ZNQ	B	2.3.1.29	GlycineC-acetyltransferase
caseinkinase,putative	1ZNQ	B	2.7.11.1	Non-specificserine/threonineproteinkinase
cystathioninebeta-synthase,cysteinesynthase,serinesulfhydrylase	1ZNQ	B	4.2.1.22	Cystathioninebeta-synthase
celldivisionproteinkinase2,cdc2-relatedproteinkinase1	1ZNQ	A	2.7.11.22	Cyclin-dependentkinase
caseinkinase,putative	1ZNQ	B	2.7.11.1	Non-specificserine/threonineproteinkinase
caseinkinase,putative	1ZNQ	A	2.7.11.1	Non-specificserine/threonineproteinkinase
celldivisionrelatedproteinkinase2,putative	1ZNQ	C	2.7.11.22	Cyclin-dependentkinase
GDP-mannose4,6dehydratase,putative	1ZNQ	A	4.2.1.47	GDP-mannose4,6-dehydratase
caseinkinase,deltaisoform,putative	1ZNQ	A	2.7.11.1	Non-specificserine/threonineproteinkinase
enoyl-CoAhydratase,mitochondrialprecursor,putative	1ZNQ	F	4.2.1.17	Enoyl-CoAhydratase
methionineaminopeptidase,putative,metallo-peptidase,clanMG,familyM24,putative	2G6P	A	3.4.11.18	Methionylaminopeptidase
methionineaminopeptidase2,putative,metallo-peptidase,clanMG,familyM24,putative	1B59	A	3.4.11.18	Methionylaminopeptidase
3-phosphoglyceratekinase,glycosomal	16PK	1	2.7.2.3	Phosphoglyceratekinase
caseinkinase,putative	1CKJ	B	2.7.11.1	Non-specificserine/threonineproteinkinase
prolineracemase,B-cellmitogenprecursor,putative	1W62	B	5.1.1.4	Prolineracemase
macrophageinfectivitypotentiator,precursor,putative,PPiase,putative,rotamase,putative,peptidyl-prolylcis-transisomerase,putative	1JVW	A	5.2.1.8	Peptidylprolylisomerase
serine/threonineproteinphosphatase,putative	1TCO	A	3.1.3.16	Phosphoproteinphosphatase
serine/threonineproteinphosphatase,putative	1AUI	A	3.1.3.16	Phosphoproteinphosphatase
methionineaminopeptidase,putative,metallo-peptidase,clanMG,familyM24,putative	2G6P	A	3.4.11.18	Methionylaminopeptidase
phosphoglyceratekinase,putative	16PK	1	2.7.2.3	Phosphoglyceratekinase
cystathioninebeta-synthase,cysteinesynthase,serinesulfhydrylase	1M54	F	4.2.1.22	Cystathioninebeta-synthase
21kDacyclophilin,putative,PPIase,putative,rotamase,putative,peptidyl-prolylcis-transisomerase,putative	1E8K	A	5.2.1.8	Peptidylprolylisomerase
ubiquitin-conjugatingenzymeE2,putative	1J7D	B	6.3.2.19	Ubiquitin--proteinligase
serine/threonineproteinphosphatasetype5,putative	1WAO	4	3.1.3.16	Phosphoproteinphosphatase
40kDacyclophilin,putative,PPIase,putative,rotamase,putative,peptidyl-prolylcis-transisomerase,putative	1IIP	A	5.2.1.8	Peptidylprolylisomerase
tyrosineaminotransferase,putative	1BW0	B	2.6.1.5	Tyrosinetransaminase
tyrosinespecificproteinphosphatase,putative	2C7S	A	3.1.3.48	Protein-tyrosine-phosphatase
serine/threonineproteinphosphatase2BcatalyticsubunitA2,putative	1TCO	A	3.1.3.16	Phosphoproteinphosphatase
tyrosineaminotransferase,putative,L-tyrosine:2-oxoglutarateaminotransferase,putative	1BW0	B	2.6.1.5	Tyrosinetransaminase
ubiquitinhydrolase,putative,cysteinepeptidase,ClanCA,familyC19,putative	2GFO	A	3.1.2.15	Ubiquitinthiolesterase
caseinkinaseII,alphachain,putative	1JWH	A	2.7.11.1	Non-specificserine/threonineproteinkinase
ubiquitin-conjugatingenzymeE2,putative	2F4W	B	6.3.2.19	Ubiquitin--proteinligase
serine/threonineproteinphosphatasetype5,putative	1WAO	4	3.1.3.16	Phosphoproteinphosphatase
adenylatekinase,putative	2AR7	A	2.7.4.3	Adenylatekinase
prolyloligopeptidase,putative,serinepeptidaseclanSC,familyS9A,putative	1QFS	A	3.4.21.26	Prolyloligopeptidase

Matching Annotation	Homologous	4	Trypanosoma_cruzi|AAHK01000056|Tc00.1047053508277.160|Annotation|GenBank|(proteincoding)(571letters)
Matching Annotation	Homologous	4	Trypanosoma_cruzi|AAHK01000060|Tc00.1047053509429.290|Annotation|GenBank|(proteincoding)(299letters)
Matching Annotation	Homologous	4	Trypanosoma_cruzi|AAHK01000073|Tc00.1047053510105.240|Annotation|GenBank|(proteincoding)(320letters)
Matching Annotation	Homologous	4	Trypanosoma_cruzi|AAHK01000082|Tc00.1047053507023.120|Annotation|GenBank|(proteincoding)(510letters)
Matching Annotation	Homologous	4	Trypanosoma_cruzi|AAHK01000082|Tc00.1047053507023.200|Annotation|GenBank|(proteincoding)(472letters)
Matching Annotation	Homologous	4	Trypanosoma_cruzi|AAHK01000090|Tc00.1047053507641.60|Annotation|GenBank|(proteincoding)(504letters)
Matching Annotation	Homologous	4	Trypanosoma_cruzi|AAHK01000094|Tc00.1047053506321.310|Annotation|GenBank|(proteincoding)(400letters)
Matching Annotation	Homologous	4	Trypanosoma_cruzi|AAHK01000096|Tc00.1047053511817.40|Annotation|GenBank|(proteincoding)(288letters)
Matching Annotation	Homologous	4	Trypanosoma_cruzi|AAHK01000102|Tc00.1047053510431.250|Annotation|GenBank|(proteincoding)(362letters)
Matching Annotation	Homologous	4	Trypanosoma_cruzi|AAHK01000104|Tc00.1047053510421.180|Annotation|GenBank|(proteincoding)(414letters)
Matching Annotation	Homologous	4	Trypanosoma_cruzi|AAHK01000107|Tc00.1047053506195.80|Annotation|GenBank|(proteincoding)(218letters)
Matching Annotation	Homologous	4	Trypanosoma_cruzi|AAHK01000113|Tc00.1047053506945.110|Annotation|GenBank|(proteincoding)(385letters)
Matching Annotation	Homologous	4	Trypanosoma_cruzi|AAHK01000113|Tc00.1047053506945.20|Annotation|GenBank|(proteincoding)(527letters)
Matching Annotation	Homologous	4	Trypanosoma_cruzi|AAHK01000119|Tc00.1047053510729.10|Annotation|GenBank|(proteincoding)(978letters)
Matching Annotation	Homologous	4	Trypanosoma_cruzi|AAHK01000123|Tc00.1047053511421.60|Annotation|GenBank|(proteincoding)(345letters)
Matching Annotation	Homologous	4	Trypanosoma_cruzi|AAHK01000129|Tc00.1047053509733.180|Annotation|GenBank|(proteincoding)(214letters)
Matching Annotation	Homologous	4	Trypanosoma_cruzi|AAHK01000135|Tc00.1047053507603.230|Annotation|GenBank|(proteincoding)(299letters)
Matching Annotation	Homologous	4	Trypanosoma_cruzi|AAHK01000138|Tc00.1047053511751.120|Annotation|GenBank|(proteincoding)(510letters)
Matching Annotation	Homologous	4	Trypanosoma_cruzi|AAHK01000138|Tc00.1047053511751.30|Annotation|GenBank|(proteincoding)(473letters)
Matching Annotation	Homologous	4	Trypanosoma_cruzi|AAHK01000154|Tc00.1047053508647.270|Annotation|GenBank|(proteincoding)(573letters)
Matching Annotation	Homologous	4	Trypanosoma_cruzi|AAHK01000154|Tc00.1047053508647.280|Annotation|GenBank|(proteincoding)(565letters)
Matching Annotation	Homologous	4	Trypanosoma_cruzi|AAHK01000167|Tc00.1047053510661.60|Annotation|GenBank|(proteincoding)(512letters)
Matching Annotation	Homologous	4	Trypanosoma_cruzi|AAHK01000183|Tc00.1047053509695.10|Annotation|GenBank|(proteincoding)(534letters)
Matching Annotation	Homologous	4	Trypanosoma_cruzi|AAHK01000187|Tc00.1047053507053.70|Annotation|GenBank|(proteincoding)(334letters)
Matching Annotation	Homologous	4	Trypanosoma_cruzi|AAHK01000233|Tc00.1047053506503.69|Annotation|GenBank|(proteincoding)(323letters)
Matching Annotation	Homologous	4	Trypanosoma_cruzi|AAHK01000235|Tc00.1047053508181.140|Annotation|GenBank|(proteincoding)(271letters)
Matching Annotation	Homologous	4	Trypanosoma_cruzi|AAHK01000242|Tc00.1047053510089.170|Annotation|GenBank|(proteincoding)(414letters)
Matching Annotation	Homologous	4	Trypanosoma_cruzi|AAHK01000248|Tc00.1047053509167.100|Annotation|GenBank|(proteincoding)(975letters)
Matching Annotation	Homologous	4	Trypanosoma_cruzi|AAHK01000250|Tc00.1047053509109.120|Annotation|GenBank|(proteincoding)(180letters)
Matching Annotation	Homologous	4	Trypanosoma_cruzi|AAHK01000250|Tc00.1047053509109.130|Annotation|GenBank|(proteincoding)(176letters)
Matching Annotation	Homologous	4	Trypanosoma_cruzi|AAHK01000253|Tc00.1047053505807.120|Annotation|GenBank|(proteincoding)(659letters)
Matching Annotation	Homologous	4	Trypanosoma_cruzi|AAHK01000266|Tc00.1047053509179.140|Annotation|GenBank|(proteincoding)(283letters)
Matching Annotation	Homologous	4	Trypanosoma_cruzi|AAHK01000271|Tc00.1047053507047.120|Annotation|GenBank|(proteincoding)(283letters)
Matching Annotation	Homologous	4	Trypanosoma_cruzi|AAHK01000273|Tc00.1047053506743.110|Annotation|GenBank|(proteincoding)(180letters)
Matching Annotation	Homologous	4	Trypanosoma_cruzi|AAHK01000273|Tc00.1047053506743.130|Annotation|GenBank|(proteincoding)(176letters)
Matching Annotation	Homologous	4	Trypanosoma_cruzi|AAHK01000278|Tc00.1047053510861.140|Annotation|GenBank|(proteincoding)(297letters)
Matching Annotation	Homologous	4	Trypanosoma_cruzi|AAHK01000282|Tc00.1047053508577.160|Annotation|GenBank|(proteincoding)(246letters)
Matching Annotation	Homologous	4	Trypanosoma_cruzi|AAHK01000283|Tc00.1047053509647.70|Annotation|GenBank|(proteincoding)(206letters)
Matching Annotation	Homologous	4	Trypanosoma_cruzi|AAHK01000305|Tc00.1047053507521.50|Annotation|GenBank|(proteincoding)(246letters)
Matching Annotation	Homologous	4	Trypanosoma_cruzi|AAHK01000330|Tc00.1047053509717.90|Annotation|GenBank|(proteincoding)(320letters)
Matching Annotation	Homologous	4	Trypanosoma_cruzi|AAHK01000332|Tc00.1047053506779.50|Annotation|GenBank|(proteincoding)(205letters)
Matching Annotation	Homologous	4	Trypanosoma_cruzi|AAHK01000342|Tc00.1047053510603.60|Annotation|GenBank|(proteincoding)(176letters)
Matching Annotation	Homologous	4	Trypanosoma_cruzi|AAHK01000342|Tc00.1047053510603.80|Annotation|GenBank|(proteincoding)(180letters)
Matching Annotation	Homologous	4	Trypanosoma_cruzi|AAHK01000344|Tc00.1047053510575.180|Annotation|GenBank|(proteincoding)(198letters)
Matching Annotation	Homologous	4	Trypanosoma_cruzi|AAHK01000348|Tc00.1047053511859.170|Annotation|GenBank|(proteincoding)(306letters)
Matching Annotation	Homologous	4	Trypanosoma_cruzi|AAHK01000349|Tc00.1047053509967.30|Annotation|GenBank|(proteincoding)(534letters)
Matching Annotation	Homologous	4	Trypanosoma_cruzi|AAHK01000425|Tc00.1047053505183.20|Annotation|GenBank|(proteincoding)(573letters)
Matching Annotation	Homologous	4	Trypanosoma_cruzi|AAHK01000425|Tc00.1047053505183.30|Annotation|GenBank|(proteincoding)(565letters)
Matching Annotation	Homologous	4	Trypanosoma_cruzi|AAHK01000453|Tc00.1047053507831.70|Annotation|GenBank|(proteincoding)(378letters)
Matching Annotation	Homologous	4	Trypanosoma_cruzi|AAHK01000472|Tc00.1047053508569.20|Annotation|GenBank|(proteincoding)(160letters)
Matching Annotation	Homologous	4	Trypanosoma_cruzi|AAHK01000478|Tc00.1047053511153.140|Annotation|GenBank|(proteincoding)(205letters)

glutaminyl-tRNAsynthetase,putative	1O0C	A	6.1.1.18	Glutamine--tRNAligase
hydroxyacylglutathionehydrolase,putative,glyoxalaseII,putative	1QH5	B	3.1.2.6	Hydroxyacylglutathionehydrolase
shortchain3-hydroxyacyl-coadehydrogenase,putative	1F14	A	1.1.1.35	3-hydroxyacyl-CoAdehydrogenase
aldehydedehydrogenasefamily,putative	1AD3	B	1.2.1.5	Aldehydedehydrogenase(NAD(P)(+))
queuinetRNA-ribosyltransferase,putative	1OZQ	A	2.4.2.29	QueuinetRNA-ribosyltransferase
aldehydedehydrogenase,putative	1O9J	D	1.2.1.3	Aldehydedehydrogenase(NAD(+))
phosphoribosylpyrophosphatesynthetase,putative	2H08	B	2.7.6.1	Ribose-phosphatediphosphokinase
NADH-cytochromeB5reductase,putative	1UMK	A	1.6.2.2	Cytochrome-b5reductase
phosphoribosylpyrophosphatesynthetase,putative	2H08	B	2.7.6.1	Ribose-phosphatediphosphokinase
caseinkinaseI,putative	1CKJ	B	2.7.11.1	Non-specificserine/threonineproteinkinase
adenylatekinase,putative	1ZIN	1	2.7.4.3	Adenylatekinase
caseinkinase1isoform2,putative	1CKJ	B	2.7.11.1	Non-specificserine/threonineproteinkinase
PAS-domaincontainingphosphoglyceratekinase,putative	16PK	1	2.7.2.3	Phosphoglyceratekinase
AMPdeaminase,putative	2A3L	A	3.5.4.6	AMPdeaminase
deoxyhypusinesynthase,putative	1DHS	1	2.5.1.46	Deoxyhypusinesynthase
adenylatekinase,putative	2EU8	A	2.7.4.3	Adenylatekinase
hydroxyacylglutathionehydrolase,putative,glyoxalaseII,putative	1QH5	B	3.1.2.6	Hydroxyacylglutathionehydrolase
aldehydedehydrogenasefamily,putative	1AD3	B	1.2.1.5	Aldehydedehydrogenase(NAD(P)(+))
queuinetRNA-ribosyltransferase,putative	1OZQ	A	2.4.2.29	QueuinetRNA-ribosyltransferase
malicenzyme,putative	2AW5	B	1.1.1.40	Malatedehydrogenase(oxaloacetate-decarboxylating)(NADP(+))
malicenzyme,putative	2AW5	C	1.1.1.40	Malatedehydrogenase(oxaloacetate-decarboxylating)(NADP(+))
glycerolkinase,glycosomal,putative	1R59	O	2.7.1.30	Glycerolkinase
aspartyl-tRNAsynthetase,putative	1ASZ	B	6.1.1.12	Aspartate--tRNAligase
geranylgeranyltransferasetypeIIbetasubunit,putative	1LTX	B	2.5.1.60	ProteingeranylgeranyltransferasetypeII
glycosomalmalatedehydrogenase,putative	2CMD	1	1.1.1.37	Malatedehydrogenase
inorganicpyrophosphatase,putative	1HUK	B	3.6.1.1	Inorganicdiphosphatase
caseinkinaseI,putative	1EH4	B	2.7.11.1	Non-specificserine/threonineproteinkinase
valyl-tRNAsynthetase,putative	1IYW	B	6.1.1.9	Valine--tRNAligase
proteintyrosinephosphatase,putative	1V3A	A	3.1.3.48	Protein-tyrosine-phosphatase
proteintyrosinephosphatase-likeprotein,putative	1XM2	F	3.1.3.48	Protein-tyrosine-phosphatase
lysyl-tRNAsynthetase,putative	1BBW	A	6.1.1.6	Lysine--tRNAligase
myo-inositol-1(or4)-monophosphatase1,putative	1IMB	B	3.1.3.25	Inositol-phosphatephosphatase
myo-inositol-1(or4)-monophosphatase1,putative	1IMB	B	3.1.3.25	Inositol-phosphatephosphatase
proteintyrosinephosphatase,putative	1X24	A	3.1.3.48	Protein-tyrosine-phosphatase
proteintyrosinephosphatase-likeprotein,putative	1X24	B	3.1.3.48	Protein-tyrosine-phosphatase
serine/threonineproteinkinase,putative,proteinkinase,putative	2C3J	A	2.7.11.1	Non-specificserine/threonineproteinkinase
proteasomebeta6subunit,putative,20Sproteasomebeta6subunit,putative	1G0U	Z	3.4.25.1	Proteasomeendopeptidasecomplex
guanylatekinase,putative	1LVG	A	2.7.4.8	Guanylatekinase
proteasomebeta6subunit,putative,20Sproteasomebeta6subunit,putative	1G0U	Z	3.4.25.1	Proteasomeendopeptidasecomplex
shortchain3-hydroxyacyl-coadehydrogenase,putative	1F14	A	1.1.1.35	3-hydroxyacyl-CoAdehydrogenase
proteasomebeta3subunit,putative	1IRU	X	3.4.25.1	Proteasomeendopeptidasecomplex
proteintyrosinephosphatase-likeprotein,putative	1XM2	D	3.1.3.48	Protein-tyrosine-phosphatase
proteintyrosinephosphatase-likeprotein,putative	1V3A	A	3.1.3.48	Protein-tyrosine-phosphatase
adenylatekinase,putative	2BWJ	D	2.7.4.3	Adenylatekinase
NADH-cytochromeB5reductase,putative	1UMK	A	1.6.2.2	Cytochrome-b5reductase
aspartyl-tRNAsynthetase,putative	1ASZ	B	6.1.1.12	Aspartate--tRNAligase
malicenzyme,putative	2AW5	A	1.1.1.40	Malatedehydrogenase(oxaloacetate-decarboxylating)(NADP(+))
malicenzyme,putative	1GQ2	C	1.1.1.40	Malatedehydrogenase(oxaloacetate-decarboxylating)(NADP(+))
pyruvatedehydrogenaseE1componentalphasubunit,putative	1NI4	C	1.2.4.1	Pyruvatedehydrogenase(acetyl-transferring)
ubiquitin-conjugatingenzymeE2,putative,ubiquitin-proteinligase,putative,ubiquitincarrierprotein,putative	2BEP	A	6.3.2.19	Ubiquitin--proteinligase
proteasomebeta3subunit,putative	1IRU	X	3.4.25.1	Proteasomeendopeptidasecomplex

Matching Annotation	Homologous	4	Trypanosoma_cruzi|AAHK01000493|Tc00.1047053509213.70|Annotation|GenBank|(proteincoding)(244letters)
Matching Annotation	Homologous	4	Trypanosoma_cruzi|AAHK01000501|Tc00.1047053506835.70|Annotation|GenBank|(proteincoding)(527letters)
Matching Annotation	Homologous	4	Trypanosoma_cruzi|AAHK01000538|Tc00.1047053510667.60|Annotation|GenBank|(proteincoding)(179letters)
Matching Annotation	Homologous	4	Trypanosoma_cruzi|AAHK01000559|Tc00.1047053510515.60|Annotation|GenBank|(proteincoding)(584letters)
Matching Annotation	Homologous	4	Trypanosoma_cruzi|AAHK01000588|Tc00.1047053506649.70|Annotation|GenBank|(proteincoding)(344letters)
Matching Annotation	Homologous	4	Trypanosoma_cruzi|AAHK01000598|Tc00.1047053509011.20|Annotation|GenBank|(proteincoding)(1731letters)
Matching Annotation	Homologous	4	Trypanosoma_cruzi|AAHK01000603|Tc00.1047053509607.10|Annotation|GenBank|(proteincoding)(160letters)
Matching Annotation	Homologous	4	Trypanosoma_cruzi|AAHK01000603|Tc00.1047053509607.70|Annotation|GenBank|(proteincoding)(297letters)
Matching Annotation	Homologous	4	Trypanosoma_cruzi|AAHK01000605|Tc00.1047053504055.81|Annotation|GenBank|(proteincoding)(225letters)
Matching Annotation	Homologous	4	Trypanosoma_cruzi|AAHK01000612|Tc00.1047053511293.69|Annotation|GenBank|(proteincoding)(323letters)
Matching Annotation	Homologous	4	Trypanosoma_cruzi|AAHK01000641|Tc00.1047053506839.60|Annotation|GenBank|(proteincoding)(668letters)
Matching Annotation	Homologous	4	Trypanosoma_cruzi|AAHK01000641|Tc00.1047053506839.80|Annotation|GenBank|(proteincoding)(390letters)
Matching Annotation	Homologous	4	Trypanosoma_cruzi|AAHK01000648|Tc00.1047053509775.40|Annotation|GenBank|(proteincoding)(233letters)
Matching Annotation	Homologous	4	Trypanosoma_cruzi|AAHK01000666|Tc00.1047053511837.70|Annotation|GenBank|(proteincoding)(417letters)
Matching Annotation	Homologous	4	Trypanosoma_cruzi|AAHK01000677|Tc00.1047053507009.100|Annotation|GenBank|(proteincoding)(179letters)
Matching Annotation	Homologous	4	Trypanosoma_cruzi|AAHK01000693|Tc00.1047053510257.30|Annotation|GenBank|(proteincoding)(244letters)
Matching Annotation	Homologous	4	Trypanosoma_cruzi|AAHK01000712|Tc00.1047053508781.110|Annotation|GenBank|(proteincoding)(431letters)
Matching Annotation	Homologous	4	Trypanosoma_cruzi|AAHK01000775|Tc00.1047053507061.30|Annotation|GenBank|(proteincoding)(233letters)
Matching Annotation	Homologous	4	Trypanosoma_cruzi|AAHK01000776|Tc00.1047053508717.10|Annotation|GenBank|(proteincoding)(668letters)
Matching Annotation	Homologous	4	Trypanosoma_cruzi|AAHK01000852|Tc00.1047053510243.50|Annotation|GenBank|(proteincoding)(198letters)
Matching Annotation	Homologous	4	Trypanosoma_cruzi|AAHK01000862|Tc00.1047053508971.30|Annotation|GenBank|(proteincoding)(580letters)
Matching Annotation	Homologous	4	Trypanosoma_cruzi|AAHK01000904|Tc00.1047053503479.30|Annotation|GenBank|(proteincoding)(260letters)
Matching Annotation	Homologous	4	Trypanosoma_cruzi|AAHK01000920|Tc00.1047053508357.40|Annotation|GenBank|(proteincoding)(557letters)
Matching Annotation	Homologous	4	Trypanosoma_cruzi|AAHK01000936|Tc00.1047053509047.110|Annotation|GenBank|(proteincoding)(458letters)
Matching Annotation	Homologous	4	Trypanosoma_cruzi|AAHK01000955|Tc00.1047053506585.70|Annotation|GenBank|(proteincoding)(629letters)
Matching Annotation	Homologous	4	Trypanosoma_cruzi|AAHK01000963|Tc00.1047053511857.80|Annotation|GenBank|(proteincoding)(791letters)
Matching Annotation	Homologous	4	Trypanosoma_cruzi|AAHK01000982|Tc00.1047053510131.50|Annotation|GenBank|(proteincoding)(604letters)
Matching Annotation	Homologous	4	Trypanosoma_cruzi|AAHK01000982|Tc00.1047053510131.70|Annotation|GenBank|(proteincoding)(514letters)
Matching Annotation	Homologous	4	Trypanosoma_cruzi|AAHK01001014|Tc00.1047053509859.40|Annotation|GenBank|(proteincoding)(584letters)
Matching Annotation	Homologous	4	Trypanosoma_cruzi|AAHK01001042|Tc00.1047053506831.70|Annotation|GenBank|(proteincoding)(600letters)
Matching Annotation	Homologous	4	Trypanosoma_cruzi|AAHK01001047|Tc00.1047053507883.100|Annotation|GenBank|(proteincoding)(374letters)
Matching Annotation	Homologous	4	Trypanosoma_cruzi|AAHK01001047|Tc00.1047053507883.80|Annotation|GenBank|(proteincoding)(218letters)
Matching Annotation	Homologous	4	Trypanosoma_cruzi|AAHK01001103|Tc00.1047053511299.70|Annotation|GenBank|(proteincoding)(362letters)
Matching Annotation	Homologous	4	Trypanosoma_cruzi|AAHK01001216|Tc00.1047053510945.70|Annotation|GenBank|(proteincoding)(418letters)
Matching Annotation	Homologous	4	Trypanosoma_cruzi|AAHK01001253|Tc00.1047053506853.50|Annotation|GenBank|(proteincoding)(431letters)
Matching Annotation	Homologous	4	Trypanosoma_cruzi|AAHK01001253|Tc00.1047053506853.50|Annotation|GenBank|(proteincoding)(431letters)
Matching Annotation	Homologous	4	Trypanosoma_cruzi|AAHK01001267|Tc00.1047053510437.40|Annotation|GenBank|(proteincoding)(332letters)
Matching Annotation	Homologous	4	Trypanosoma_cruzi|AAHK01001294|Tc00.1047053503755.10|Annotation|GenBank|(proteincoding)(597letters)
Matching Annotation	Homologous	4	Trypanosoma_cruzi|AAHK01001294|Tc00.1047053503755.30|Annotation|GenBank|(proteincoding)(514letters)
Matching Annotation	Homologous	4	Trypanosoma_cruzi|AAHK01001313|Tc00.1047053511047.40|Annotation|GenBank|(proteincoding)(306letters)
Matching Annotation	Homologous	4	Trypanosoma_cruzi|AAHK01001320|Tc00.1047053503841.70|Annotation|GenBank|(proteincoding)(404letters)
Matching Annotation	Homologous	4	Trypanosoma_cruzi|AAHK01001356|Tc00.1047053510123.20|Annotation|GenBank|(proteincoding)(417letters)
Matching Annotation	Homologous	4	Trypanosoma_cruzi|AAHK01001761|Tc00.1047053503687.40|Annotation|GenBank|(proteincoding)(354letters)
Matching Annotation	Homologous	4	Trypanosoma_cruzi|AAHK01001830|Tc00.1047053508351.10|Annotation|GenBank|(proteincoding)(344letters)
Matching Annotation	Homologous	4	Trypanosoma_cruzi|AAHK01001847|Tc00.1047053503851.24|Annotation|GenBank|(proteincoding)(176letters)
Matching Annotation	Homologous	4	Trypanosoma_cruzi|AAHK01001900|Tc00.1047053503581.20|Annotation|GenBank|(proteincoding)(324letters)
Matching Annotation	Homologous	4	Trypanosoma_cruzi|AAHK01001953|Tc00.1047053506937.10|Annotation|GenBank|(proteincoding)(332letters)
Matching Annotation	Homologous	4	Trypanosoma_cruzi|AAHK01002055|Tc00.1047053503471.10|Annotation|GenBank|(proteincoding)(180letters)
Matching Annotation	Homologous	4	Trypanosoma_cruzi|AAHK01002070|Tc00.1047053503513.10|Annotation|GenBank|(proteincoding)(408letters)
Matching Annotation	Homologous	4	Trypanosoma_cruzi|AAHK01002153|Tc00.1047053504167.30|Annotation|GenBank|(proteincoding)(352letters)
Matching Annotation	Homologous	4	Trypanosoma_cruzi|AAHK01002181|Tc00.1047053510221.39|Annotation|GenBank|(proteincoding)(712letters)

ribulose-5-phosphate3-epimerase,putative	1H1Z	B	5.1.3.1	Ribulose-phosphate3-epimerase
PAS-domaincontainingphosphoglyceratekinase,putative	16PK	1	2.7.2.3	Phosphoglyceratekinase
peptidyl-prolylcis-transisomerase(cyclophilin),putative,PPIase,putative,rotamase,putative,peptidyl-prolylcis-transisomerase,putative	1XYH	A	5.2.1.8	Peptidylprolylisomerase
cytosolicleucylaminopeptidase,putative,metallo-peptidase,clanMF,familyM17,putative	1GYT	L	3.4.11.1	Leucylaminopeptidase
fructose-1,6-bisphosphatase,cytosolic,putative	2FIE	D	3.1.3.11	Fructose-bisphosphatase
RNApolymeraseIIAlargestsubunit,putative	1I3Q	A	2.7.7.6	DNA-directedRNApolymerase
ubiquitin-conjugatingenzymeE2,putative,ubiquitin-proteinligase,putative,ubiquitincarrierprotein,putative	2BEP	A	6.3.2.19	Ubiquitin--proteinligase
serine/threonineproteinkinase,putative,proteinkinase,putative	2CGV	A	2.7.11.1	Non-specificserine/threonineproteinkinase
ubiquitin-conjugatingenzymeE2,putative	2F4W	B	6.3.2.19	Ubiquitin--proteinligase
glycosomalmalatedehydrogenase,putative	2CMD	1	1.1.1.37	Malatedehydrogenase
tyrosinespecificproteinphosphatase,putative	1LAR	B	3.1.3.48	Protein-tyrosine-phosphatase
phosphoribosylpyrophosphatesynthetase,putative	2H07	B	2.7.6.1	Ribose-phosphatediphosphokinase
ironsuperoxidedismutase,putative	2CE4	A	1.15.1.1	Superoxidedismutase
mitogenactivatedproteinkinase,putative	4ERK	1	2.7.11.24	Mitogen-activatedproteinkinase
cyclophilin,putative,PPIase,putative,rotamase,putative,peptidyl-prolylcis-transisomerase,putative	1XYH	A	5.2.1.8	Peptidylprolylisomerase
ribulose-5-phosphate3-epimerase,putative	1H1Z	B	5.1.3.1	Ribulose-phosphate3-epimerase
2-oxoisovaleratedehydrogenasealphasubunit,putative	2BEW	A	1.2.4.4	3-methyl-2-oxobutanoatedehydrogenase(2-methylpropanoyl-transferring)
ironsuperoxidedismutase,putative	2CE4	A	1.15.1.1	Superoxidedismutase
tyrosinespecificproteinphosphatase,putative	1LAR	B	3.1.3.48	Protein-tyrosine-phosphatase
adenylatekinase,putative	2BWJ	F	2.7.4.3	Adenylatekinase
lysyl-tRNAsynthetase,putative	1BBW	A	6.1.1.6	Lysine--tRNAligase
adenylatekinase,putative	2AR7	A	2.7.4.3	Adenylatekinase
ubiquitinhydrolase,putative,cysteinepeptidase,ClanCA,familyC19,putative	2GFO	A	3.1.2.15	Ubiquitinthiolesterase
racserine-threoninekinase,putative,proteinkinase,putative	2ESM	B	2.7.11.1	Non-specificserine/threonineproteinkinase
P450reductase,putative	1JA1	A	1.6.2.4	NADPH--hemoproteinreductase
AMPdeaminase,putative	2A3L	A	3.5.4.6	AMPdeaminase
DNApolymerasekappa,putative,DNApolymeraseIV,putative	1T94	B	2.7.7.7	DNA-directedDNApolymerase
DNApolymerasekappa,putative,DNApolymeraseIV,putative	1T94	B	2.7.7.7	DNA-directedDNApolymerase
cytosolicleucylaminopeptidase,putative,metallo-peptidase,clanMF,familyM17,putative	1GYT	L	3.4.11.1	Leucylaminopeptidase
glutamyl-tRNAsynthetase,putative	1O0C	A	6.1.1.18	Glutamine--tRNAligase
malatedehydrogenase,putative	1SEV	A	1.1.1.37	Malatedehydrogenase
adenylatekinase,putative	1P3J	A	2.7.4.3	Adenylatekinase
mitogen-activatedproteinkinase,putative	2FYS	A	2.7.11.24	Mitogen-activatedproteinkinase
aspartateaminotransferase,mitochondrial,putative	9AAT	B	2.6.1.1	Aspartatetransaminase
2-oxoisovaleratedehydrogenasealphasubunit,putative	2BEW	A	1.2.4.1	3-methyl-2-oxobutanoatedehydrogenase(2-methylpropanoyl-transferring)
2-oxoisovaleratedehydrogenasealphasubunit,putative	2BEW	A	1.2.4.4	3-methyl-2-oxobutanoatedehydrogenase(2-methylpropanoyl-transferring)
cytosolicmalatedehydrogenase,putative	5MDH	A	1.1.1.37	Malatedehydrogenase
DNApolymerasekappa,putative,DNApolymeraseIV,putative	1T94	B	2.7.7.7	DNA-directedDNApolymerase
DNApolymerasekappa,putative,DNApolymeraseIV,putative	1T94	B	2.7.7.7	DNA-directedDNApolymerase
NADH-cytochromeB5reductase,putative	1UMK	A	1.6.2.2	Cytochrome-b5reductase
aspartateaminotransferase,putative	1AKA	A	2.6.1.1	Aspartatetransaminase
mitogenactivatedproteinkinase,putative	4ERK	1	2.7.11.24	Mitogen-activatedproteinkinase
40kDacyclophilin,putative,PPIase,putative,rotamase,putative,peptidyl-prolylcis-transisomerase,putative	1IIP	A	5.2.1.8	Peptidylprolylisomerase
fructose-1,6-bisphosphatase,cytosolic,putative	2F3B	A	3.1.3.11	Fructose-bisphosphatase
proteintyrosinephosphatase-likieprotein,putative	1XM2	F	3.1.3.48	Protein-tyrosine-phosphatase
cyclophilin,putative,PPIase,putative,rotamase,putative,peptidyl-prolylcis-transisomerase,putative	1IIP	A	5.2.1.8	Peptidylprolylisomerase
cytosolicmalatedehydrogenase,putative	5MDH	A	1.1.1.37	Malatedehydrogenase
proteintyrosinephosphatase,putative	1X24	A	3.1.3.48	Protein-tyrosine-phosphatase
caseinkinaseII,alphachain,putative	1JWH	A	2.7.11.1	Non-specificserine/threonineproteinkinase
proteinkinase,putative	1ZZL	A	2.7.11.24	Mitogen-activatedproteinkinase
adenosinemonophosphatedeaminase,putative	2A3L	A	3.5.4.6	AMPdeaminase

Matching Annotation	Homologous	4	Trypanosoma_cruzi|AAHK01002214|Tc00.1047053511691.10|Annotation|GenBank|(proteincoding)(396letters)
Matching Annotation	Homologous	4	Trypanosoma_cruzi|AAHK01002222|Tc00.1047053503815.20|Annotation|GenBank|(proteincoding)(652letters)
Matching Annotation	Homologous	4	Trypanosoma_cruzi|AAHK01002265|Tc00.1047053503679.10|Annotation|GenBank|(proteincoding)(404letters)
Matching Annotation	Homologous	4	Trypanosoma_cruzi|AAHK01002274|Tc00.1047053507107.40|Annotation|GenBank|(proteincoding)(352letters)
Matching Annotation	Homologous	4	Trypanosoma_cruzi|AAHK01002321|Tc00.1047053510671.20|Annotation|GenBank|(proteincoding)(371letters)
Matching Annotation	Homologous	4	Trypanosoma_cruzi|AAHK01002408|Tc00.1047053503753.29|Annotation|GenBank|(proteincoding)(249letters)
Matching Annotation	Homologous	4	Trypanosoma_cruzi|AAHK01003028|Tc00.1047053510777.20|Annotation|GenBank|(proteincoding)(564letters)
Matching Annotation	Homologous	4	Trypanosoma_cruzi|AAHK01003840|Tc00.1047053508129.9|Annotation|GenBank|(proteincoding)(821letters)
Matching Annotation	Homologous	4	Trypanosoma_cruzi|AAHK01003980|Tc00.1047053508795.19|Annotation|GenBank|(proteincoding)(401letters)
Matching Annotation	Homologous	4	Trypanosoma_cruzi|AAHK01005134|Tc00.1047053461927.9|Annotation|GenBank|(proteincoding)(563letters)
Matching Annotation	Homologous	5	Trypanosoma_cruzi|AAHK01000001|Tc00.1047053508153.820|Annotation|GenBank|(proteincoding)(276letters)
Matching Annotation	Homologous	5	Trypanosoma_cruzi|AAHK01000006|Tc00.1047053506559.524|Annotation|GenBank|(proteincoding)(316letters)
Matching Annotation	Homologous	5	Trypanosoma_cruzi|AAHK01000006|Tc00.1047053506559.530|Annotation|GenBank|(proteincoding)(286letters)
Matching Annotation	Homologous	5	Trypanosoma_cruzi|AAHK01000017|Tc00.1047053511127.400|Annotation|GenBank|(proteincoding)(666letters)
Matching Annotation	Homologous	5	Trypanosoma_cruzi|AAHK01000028|Tc00.1047053506435.30|Annotation|GenBank|(proteincoding)(927letters)
Matching Annotation	Homologous	5	Trypanosoma_cruzi|AAHK01000081|Tc00.1047053504105.30|Annotation|GenBank|(proteincoding)(1553letters)
Matching Annotation	Homologous	5	Trypanosoma_cruzi|AAHK01000099|Tc00.1047053511727.300|Annotation|GenBank|(proteincoding)(317letters)
Matching Annotation	Homologous	5	Trypanosoma_cruzi|AAHK01000102|Tc00.1047053510431.140|Annotation|GenBank|(proteincoding)(461letters)
Matching Annotation	Homologous	5	Trypanosoma_cruzi|AAHK01000107|Tc00.1047053506195.90|Annotation|GenBank|(proteincoding)(241letters)
Matching Annotation	Homologous	5	Trypanosoma_cruzi|AAHK01000248|Tc00.1047053509167.120|Annotation|GenBank|(proteincoding)(370letters)
Matching Annotation	Homologous	5	Trypanosoma_cruzi|AAHK01000252|Tc00.1047053507723.189|Annotation|GenBank|(proteincoding)(181letters)
Matching Annotation	Homologous	5	Trypanosoma_cruzi|AAHK01000262|Tc00.1047053511825.210|Annotation|GenBank|(proteincoding)(241letters)
Matching Annotation	Homologous	5	Trypanosoma_cruzi|AAHK01000296|Tc00.1047053506821.210|Annotation|GenBank|(proteincoding)(308letters)
Matching Annotation	Homologous	5	Trypanosoma_cruzi|AAHK01000365|Tc00.1047053509029.10|Annotation|GenBank|(proteincoding)(418letters)
Matching Annotation	Homologous	5	Trypanosoma_cruzi|AAHK01000366|Tc00.1047053503893.70|Annotation|GenBank|(proteincoding)(325letters)
Matching Annotation	Homologous	5	Trypanosoma_cruzi|AAHK01000468|Tc00.1047053511491.100|Annotation|GenBank|(proteincoding)(382letters)
Matching Annotation	Homologous	5	Trypanosoma_cruzi|AAHK01000532|Tc00.1047053509911.90|Annotation|GenBank|(proteincoding)(1071letters)
Matching Annotation	Homologous	5	Trypanosoma_cruzi|AAHK01000604|Tc00.1047053509901.170|Annotation|GenBank|(proteincoding)(401letters)
Matching Annotation	Homologous	5	Trypanosoma_cruzi|AAHK01000611|Tc00.1047053507601.10|Annotation|GenBank|(proteincoding)(637letters)
Matching Annotation	Homologous	5	Trypanosoma_cruzi|AAHK01000734|Tc00.1047053508273.10|Annotation|GenBank|(proteincoding)(901letters)
Matching Annotation	Homologous	5	Trypanosoma_cruzi|AAHK01000754|Tc00.1047053506947.90|Annotation|GenBank|(proteincoding)(260letters)
Matching Annotation	Homologous	5	Trypanosoma_cruzi|AAHK01000817|Tc00.1047053506221.30|Annotation|GenBank|(proteincoding)(397letters)
Matching Annotation	Homologous	5	Trypanosoma_cruzi|AAHK01000886|Tc00.1047053511237.10|Annotation|GenBank|(proteincoding)(686letters)
Matching Annotation	Homologous	5	Trypanosoma_cruzi|AAHK01000912|Tc00.1047053510749.40|Annotation|GenBank|(proteincoding)(465letters)
Matching Annotation	Homologous	5	Trypanosoma_cruzi|AAHK01000919|Tc00.1047053504013.110|Annotation|GenBank|(proteincoding)(437letters)
Matching Annotation	Homologous	5	Trypanosoma_cruzi|AAHK01001047|Tc00.1047053507883.90|Annotation|GenBank|(proteincoding)(226letters)
Matching Annotation	Homologous	5	Trypanosoma_cruzi|AAHK01001123|Tc00.1047053506569.10|Annotation|GenBank|(proteincoding)(464letters)
Matching Annotation	Homologous	5	Trypanosoma_cruzi|AAHK01001160|Tc00.1047053509215.10|Annotation|GenBank|(proteincoding)(1029letters)
Matching Annotation	Homologous	5	Trypanosoma_cruzi|AAHK01001204|Tc00.1047053511481.50|Annotation|GenBank|(proteincoding)(251letters)
Matching Annotation	Homologous	5	Trypanosoma_cruzi|AAHK01001300|Tc00.1047053507389.70|Annotation|GenBank|(proteincoding)(1069letters)
Matching Annotation	Homologous	5	Trypanosoma_cruzi|AAHK01001467|Tc00.1047053504443.10|Annotation|GenBank|(proteincoding)(464letters)
Matching Annotation	Homologous	5	Trypanosoma_cruzi|AAHK01001472|Tc00.1047053511543.60|Annotation|GenBank|(proteincoding)(225letters)
Matching Annotation	Homologous	5	Trypanosoma_cruzi|AAHK01001482|Tc00.1047053504005.10|Annotation|GenBank|(proteincoding)(251letters)
Matching Annotation	Homologous	5	Trypanosoma_cruzi|AAHK01001766|Tc00.1047053506411.10|Annotation|GenBank|(proteincoding)(686letters)
Matching Annotation	Homologous	5	Trypanosoma_cruzi|AAHK01001797|Tc00.1047053510259.6|Annotation|GenBank|(proteincoding)(833letters)
Matching Annotation	Homologous	5	Trypanosoma_cruzi|AAHK01002373|Tc00.1047053506701.10|Annotation|GenBank|(proteincoding)(510letters)
Matching Annotation	Homologous	5	Trypanosoma_cruzi|AAHK01002542|Tc00.1047053509379.10|Annotation|GenBank|(proteincoding)(510letters)
Matching Annotation	Homologous	5	Trypanosoma_cruzi|AAHK01003711|Tc00.1047053503873.10|Annotation|GenBank|(proteincoding)(308letters)
Matching Annotation	Homologous	5	Trypanosoma_cruzi|AAHK01008020|Tc00.1047053443397.9|Annotation|GenBank|(proteincoding)(296letters)
Matching Annotation	Homologous	6	Trypanosoma_cruzi|AAHK01000006|Tc00.1047053506559.350|Annotation|GenBank|(proteincoding)(745letters)
Matching Annotation	Homologous	6	Trypanosoma_cruzi|AAHK01000010|Tc00.1047053508461.400|Annotation|GenBank|(proteincoding)(334letters)

cystathioninebeta-synthase,cysteinesynthase,serinesulfhydrylase	1M54	F	4.2.1.22	Cystathioninebeta-synthase
lysyl-tRNAsynthetase,putative	1BBW	A	6.1.1.6	Lysine--tRNAligase
aspartateaminotransferase,putative	2CST	B	2.6.1.1	Aspartatetransaminase
3,2-trans-enoyl-CoAisomerase,mitochondrialprecursor,putative	1SG4	C	5.3.3.8	Dodecenoyl-CoAisomerase
tryptophanyl-tRNAsynthetase,putative	1O5T	A	6.1.1.2	Tryptophan--tRNAligase
NADPH--cytochromep450reductase,putative	1B1C	A	1.6.2.4	NADPH--hemoproteinreductase
aspartyl-tRNAsynthetase,putative	1ASZ	B	6.1.1.12	Aspartate--tRNAligase
adenosinemonophosphatedeaminase,putative	2A3L	A	3.5.4.6	AMPdeaminase
proteinkinase,putative	1ZYD	B	2.7.11.1	Non-specificserine/threonineproteinkinase
adenosinemonophosphatedeaminase,putative	2A3L	A	3.5.4.6	AMPdeaminase
inorganicpyrophosphatase,putative	1HUJ	B	3.6.1.1	Inorganicdiphosphatase
proteinphosphatase2C,putative	2IQ1	A	3.1.3.16	Phosphoproteinphosphatase
proteinphosphatase2C,putative	2IQ1	A	3.1.3.16	Phosphoproteinphosphatase
serine/threonineproteinphosphatase,putative	1WAO	4	3.1.3.16	Phosphoproteinphosphatase
mitochondrialDNApolymeraseIproteinA,putative	1TAQ	1	2.7.7.7	DNA-directedDNApolymerase
DNA-directedRNApolymeraseIIIlargestsubunit,putative	2A69	D	2.7.7.6	DNA-directedRNApolymerase
GDP-L-fucosesynthetase,putative	1BWS	A	1.1.1.271	GDP-L-fucosesynthase
proteinkinase,putative	1Z57	A	2.7.12.1	Dual-specificitykinase
adenylatekinase,putative	2C9Y	A	2.7.4.3	Adenylatekinase
S-adenosylmethioninedecarboxylaseproenzyme,putative	1MHM	A	4.1.1.50	Adenosylmethioninedecarboxylase
proteindisulfideisomerase,putative	2DMM	A	5.3.4.1	Proteindisulfide-isomerase
dephospho-CoAkinase,putative	1VHL	A	2.7.1.24	Dephospho-CoAkinase
cytochrome-B5reductase,putative	1IB0	A	1.6.2.2	Cytochrome-b5reductase
serine/threonineproteinphosphatase,putative	1WAO	3	3.1.3.16	Phosphoproteinphosphatase
reductase,putative	1UMK	A	1.6.2.2	Cytochrome-b5reductase
phosphatase2C,putative	1A6Q	1	3.1.3.16	Phosphoproteinphosphatase
peptidase,putative,metallo-peptidase,ClanME,FamilyM16,putative	2G56	B	3.4.24.56	Insulysin
apurinic/apyrimidinicendonuclease,APE1/exoIII-relatedapurinic/apyrimidinicendonuclease	1HD7	A	4.2.99.18	DNA-(apurinicorapyrimidinicsite)lyase
phosphoproteinphosphatase,putative	1AUI	A	3.1.3.16	Phosphoproteinphosphatase
AMPdeaminase,putative,adenosinemonophosphatedeaminase-likeprotein,putative	2A3L	A	3.5.4.6	AMPdeaminase
adenylatekinase,putative	1ZAK	B	2.7.4.3	Adenylatekinase
proteinphosphatase2C,putative	1A6Q	1	3.1.3.16	Phosphoproteinphosphatase
thimetoligopeptidase,putative,metallo-peptidase,clanMA(E),familyM3,putative	1I1I	P	3.4.24.16	Neurolysin
ubiquitinhydrolase,putative,cysteinepeptidase,ClanCA,familyC19,putative	2F1Z	B	3.1.2.15	Ubiquitinthiolesterase
serine/threonine-proteinphosphatase2A,catalyticsubunit,putative	1WAO	4	3.1.3.16	Phosphoproteinphosphatase
adenylatekinase,putative	1ZAK	A	2.7.4.3	Adenylatekinase
ubiquitinhydrolase,putative,cysteinepeptidase,ClanCA,familyC19,putative	2AYO	A	3.1.2.15	Ubiquitinthiolesterase
DNApolymerasedeltacatalyticsubunit,putative	1WNS	A	2.7.7.7	DNA-directedDNApolymerase
endonucleaseIII,putative	2ABK	1	4.2.99.18	DNA-(apurinicorapyrimidinicsite)lyase
peptidase,putative,metallo-peptidase,ClanME,FamilyM16,putative	2G56	B	3.4.24.56	Insulysin
ubiquitinhydrolase,putative,cysteinepeptidase,ClanCA,familyC19,putative	2AYO	A	3.1.2.15	Ubiquitinthiolesterase
glutathioneperoxidase,putative	2F8A	B	1.11.1.9	Glutathioneperoxidase
endonucleaseIII,putative	2ABK	1	4.2.99.18	DNA-(apurinicorapyrimidinicsite)lyase
thimetoligopeptidase,putative,metallo-peptidase,clanMA(E),familyM3,putative	1I1I	P	3.4.24.16	Neurolysin
DNApolymerasedeltacatalyticsubunit,putative	1QHT	A	2.7.7.7	DNA-directedDNApolymerase
dihydrolipoamidedehydrogenase,putative	2A8X	B	1.8.1.4	Dihydrolipoyldehydrogenase
dihydrolipoamidedehydrogenase,putative	2A8X	B	1.8.1.4	Dihydrolipoyldehydrogenase
cytochrome-B5reductase,putative	1IB0	A	1.6.2.2	Cytochrome-b5reductase
NADH-cytochromeb5reductase,putative	1UMK	A	1.6.2.2	Cytochrome-b5reductase
asparaginyl-tRNAsynthetase,putative	1X56	A	6.1.1.22	Asparagine--tRNAligase
nucleosidediphosphatekinase,putative	1XQI	A	2.7.4.6	Nucleoside-diphosphatekinase

Matching Annotation	Homologous	6	Trypanosoma_cruzi|AAHK01000054|Tc00.1047053506247.10|Annotation|GenBank|(proteincoding)(209letters)
Matching Annotation	Homologous	6	Trypanosoma_cruzi|AAHK01000072|Tc00.1047053506789.180|Annotation|GenBank|(proteincoding)(653letters)
Matching Annotation	Homologous	6	Trypanosoma_cruzi|AAHK01000093|Tc00.1047053511367.260|Annotation|GenBank|(proteincoding)(655letters)
Matching Annotation	Homologous	6	Trypanosoma_cruzi|AAHK01000099|Tc00.1047053511727.40|Annotation|GenBank|(proteincoding)(247letters)
Matching Annotation	Homologous	6	Trypanosoma_cruzi|AAHK01000113|Tc00.1047053506945.220|Annotation|GenBank|(proteincoding)(716letters)
Matching Annotation	Homologous	6	Trypanosoma_cruzi|AAHK01000146|Tc00.1047053506859.10|Annotation|GenBank|(proteincoding)(224letters)
Matching Annotation	Homologous	6	Trypanosoma_cruzi|AAHK01000197|Tc00.1047053506265.30|Annotation|GenBank|(proteincoding)(924letters)
Matching Annotation	Homologous	6	Trypanosoma_cruzi|AAHK01000225|Tc00.1047053506739.190|Annotation|GenBank|(proteincoding)(678letters)
Matching Annotation	Homologous	6	Trypanosoma_cruzi|AAHK01000253|Tc00.1047053505807.10|Annotation|GenBank|(proteincoding)(266letters)
Matching Annotation	Homologous	6	Trypanosoma_cruzi|AAHK01000274|Tc00.1047053507083.30|Annotation|GenBank|(proteincoding)(522letters)
Matching Annotation	Homologous	6	Trypanosoma_cruzi|AAHK01000299|Tc00.1047053506871.100|Annotation|GenBank|(proteincoding)(260letters)
Matching Annotation	Homologous	6	Trypanosoma_cruzi|AAHK01000472|Tc00.1047053508569.130|Annotation|GenBank|(proteincoding)(749letters)
Matching Annotation	Homologous	6	Trypanosoma_cruzi|AAHK01000552|Tc00.1047053508177.129|Annotation|GenBank|(proteincoding)(275letters)
Matching Annotation	Homologous	6	Trypanosoma_cruzi|AAHK01000586|Tc00.1047053508699.10|Annotation|GenBank|(proteincoding)(1476letters)
Matching Annotation	Homologous	6	Trypanosoma_cruzi|AAHK01000716|Tc00.1047053510173.90|Annotation|GenBank|(proteincoding)(604letters)
Matching Annotation	Homologous	6	Trypanosoma_cruzi|AAHK01000752|Tc00.1047053503733.20|Annotation|GenBank|(proteincoding)(749letters)
Matching Annotation	Homologous	6	Trypanosoma_cruzi|AAHK01000771|Tc00.1047053503723.90|Annotation|GenBank|(proteincoding)(239letters)
Matching Annotation	Homologous	6	Trypanosoma_cruzi|AAHK01000778|Tc00.1047053509033.30|Annotation|GenBank|(proteincoding)(264letters)
Matching Annotation	Homologous	6	Trypanosoma_cruzi|AAHK01000819|Tc00.1047053509203.70|Annotation|GenBank|(proteincoding)(1484letters)
Matching Annotation	Homologous	6	Trypanosoma_cruzi|AAHK01000868|Tc00.1047053508865.4|Annotation|GenBank|(proteincoding)(732letters)
Matching Annotation	Homologous	6	Trypanosoma_cruzi|AAHK01000890|Tc00.1047053506315.90|Annotation|GenBank|(proteincoding)(678letters)
Matching Annotation	Homologous	6	Trypanosoma_cruzi|AAHK01001067|Tc00.1047053506483.69|Annotation|GenBank|(proteincoding)(252letters)
Matching Annotation	Homologous	6	Trypanosoma_cruzi|AAHK01001203|Tc00.1047053511499.50|Annotation|GenBank|(proteincoding)(755letters)
Matching Annotation	Homologous	6	Trypanosoma_cruzi|AAHK01001211|Tc00.1047053506025.60|Annotation|GenBank|(proteincoding)(404letters)
Matching Annotation	Homologous	6	Trypanosoma_cruzi|AAHK01001230|Tc00.1047053509721.60|Annotation|GenBank|(proteincoding)(592letters)
Matching Annotation	Homologous	6	Trypanosoma_cruzi|AAHK01001336|Tc00.1047053509505.10|Annotation|GenBank|(proteincoding)(263letters)
Matching Annotation	Homologous	6	Trypanosoma_cruzi|AAHK01001540|Tc00.1047053507897.30|Annotation|GenBank|(proteincoding)(504letters)
Matching Annotation	Homologous	6	Trypanosoma_cruzi|AAHK01001823|Tc00.1047053511735.60|Annotation|GenBank|(proteincoding)(344letters)
Matching Annotation	Homologous	6	Trypanosoma_cruzi|AAHK01001958|Tc00.1047053509261.30|Annotation|GenBank|(proteincoding)(398letters)
Matching Annotation	Homologous	6	Trypanosoma_cruzi|AAHK01002564|Tc00.1047053511521.30|Annotation|GenBank|(proteincoding)(345letters)
Matching Annotation	Homologous	6	Trypanosoma_cruzi|AAHK01002617|Tc00.1047053507693.5|Annotation|GenBank|(proteincoding)(252letters)
Matching Annotation	Homologous	6	Trypanosoma_cruzi|AAHK01002874|Tc00.1047053503935.20|Annotation|GenBank|(proteincoding)(392letters)
Matching Annotation	Homologous	6	Trypanosoma_cruzi|AAHK01002894|Tc00.1047053511813.20|Annotation|GenBank|(proteincoding)(223letters)
Matching Annotation	Homologous	6	Trypanosoma_cruzi|AAHK01002991|Tc00.1047053508585.29|Annotation|GenBank|(proteincoding)(207letters)
Matching Annotation	Homologous	6	Trypanosoma_cruzi|AAHK01004548|Tc00.1047053508537.10|Annotation|GenBank|(proteincoding)(246letters)
Matching Annotation	Homologous	7	Trypanosoma_cruzi|AAHK01000005|Tc00.1047053511277.20|Annotation|GenBank|(proteincoding)(219letters)
Matching Annotation	Homologous	7	Trypanosoma_cruzi|AAHK01000012|Tc00.1047053506885.390|Annotation|GenBank|(proteincoding)(1429letters)
Matching Annotation	Homologous	7	Trypanosoma_cruzi|AAHK01000038|Tc00.1047053508707.180|Annotation|GenBank|(proteincoding)(1207letters)
Matching Annotation	Homologous	7	Trypanosoma_cruzi|AAHK01000070|Tc00.1047053507063.100|Annotation|GenBank|(proteincoding)(629letters)
Matching Annotation	Homologous	7	Trypanosoma_cruzi|AAHK01000093|Tc00.1047053511367.70|Annotation|GenBank|(proteincoding)(269letters)
Matching Annotation	Homologous	7	Trypanosoma_cruzi|AAHK01000100|Tc00.1047053506357.90|Annotation|GenBank|(proteincoding)(219letters)
Matching Annotation	Homologous	7	Trypanosoma_cruzi|AAHK01000119|Tc00.1047053510729.299|Annotation|GenBank|(proteincoding)(190letters)
Matching Annotation	Homologous	7	Trypanosoma_cruzi|AAHK01000258|Tc00.1047053508387.120|Annotation|GenBank|(proteincoding)(659letters)
Matching Annotation	Homologous	7	Trypanosoma_cruzi|AAHK01000262|Tc00.1047053511825.220|Annotation|GenBank|(proteincoding)(959letters)
Matching Annotation	Homologous	7	Trypanosoma_cruzi|AAHK01000400|Tc00.1047053510879.10|Annotation|GenBank|(proteincoding)(179letters)
Matching Annotation	Homologous	7	Trypanosoma_cruzi|AAHK01000444|Tc00.1047053506733.20|Annotation|GenBank|(proteincoding)(1522letters)
Matching Annotation	Homologous	7	Trypanosoma_cruzi|AAHK01000583|Tc00.1047053506375.10|Annotation|GenBank|(proteincoding)(247letters)
Matching Annotation	Homologous	7	Trypanosoma_cruzi|AAHK01000628|Tc00.1047053510241.120|Annotation|GenBank|(proteincoding)(366letters)
Matching Annotation	Homologous	7	Trypanosoma_cruzi|AAHK01000645|Tc00.1047053509073.30|Annotation|GenBank|(proteincoding)(1207letters)
Matching Annotation	Homologous	7	Trypanosoma_cruzi|AAHK01000767|Tc00.1047053508995.40|Annotation|GenBank|(proteincoding)(726letters)
Matching Annotation	Homologous	7	Trypanosoma_cruzi|AAHK01000772|Tc00.1047053510579.20|Annotation|GenBank|(proteincoding)(366letters)

proteindisulfideisomerase,putative	2DMM	A	5.3.4.1	Proteindisulfide-isomerase
glutaminylcyclase,putative	2AFZ	B	2.3.2.5	Glutaminyl-peptidecyclotransferase
ubiquitinhydrolase,putative,cysteinepeptidase,ClanCA,familyC19,putative	2GFO	A	3.1.2.15	Ubiquitinthiolesterase
ubiquitin-conjugatingenzymeprotein,putative	1ZDN	A	6.3.2.19	Ubiquitin--proteinligase
serine/arginine-richproteinspecifickinaseSRPK,putative,proteinkinase,putative	1WBP	A	2.7.11.1	Non-specificserine/threonineproteinkinase
ubiquitin-conjugatingenzymeE2,putative	2AAK	1	6.3.2.19	Ubiquitin--proteinligase
mitochondrialDNApolymeraseIproteinA,putative	2KZZ	A	2.7.7.7	DNA-directedDNApolymerase
serine/threonineproteinkinase,putative,proteinkinase,putative	2EXE	A	2.7.12.1	Dual-specificitykinase
cyclophilin,putative	1XYH	A	5.2.1.8	Peptidylprolylisomerase
apurinic/apyrimidinicendonuclease,putative,APE1/exoIII-relatedapurinic/apyrimidinicendonuclease,putative	1HD7	A	4.2.99.18	DNA-(apurinicorapyrimidinicsite)lyase
polypeptidedeformylase-likeprotein,putative	1S17	B	3.5.1.88	Peptidedeformylase
6-phosphofructo-2-kinase/fructose-2,6-biphosphatase,putative	2AXN	A	2.7.1.105	6-phosphofructo-2-kinase
cystathioninebeta-synthase,putative	1M54	F	4.2.1.22	Cystathioninebeta-synthase
DNAtopoisomeraseII,putative	1BJT	1	5.99.1.3	DNAtopoisomerase(ATP-hydrolyzing)
poly(ADP-ribose)polymerase,putative	3PAX	1	2.4.2.30	NAD(+)ADP-ribosyltransferase
6-phosphofructo-2-kinase/fructose-2,6-biphosphatase,putative	2AXN	A	2.7.1.105	6-phosphofructo-2-kinase
glyceraldehyde3-phosphatedehydrogenase,C-terminaldomain,putative	1DC4	A	1.2.1.12	Glyceraldehyde-3-phosphatedehydrogenase(phosphorylating)
cyclophilin,putative	1XYH	A	5.2.1.8	Peptidylprolylisomerase
DNAtopoisomeraseII,putative	1BJT	1	5.99.1.3	DNAtopoisomerase(ATP-hydrolyzing)
proteinphosphatase2C,putative	1A6Q	1	3.1.3.16	Phosphoproteinphosphatase
serine/threonineproteinkinase,putative,proteinkinase,putative	2EXE	A	2.7.12.1	Dual-specificitykinase
mitogen-activatedproteinkinase,putative	2FYS	A	2.7.11.24	Mitogen-activatedproteinkinase
ubiquitinhydrolase,putative,cysteinepeptidase,ClanCA,familyC19,putative	2GFO	A	3.1.2.15	Ubiquitinthiolesterase
2-oxoglutaratedehydrogenase,E2component,dihydrolipoamidesuccinyltransferase,putative	1SCZ	A	2.3.1.61	Dihydrolipoyllysine-residuesuccinyltransferase
poly(ADP-ribose)polymerase,putative	3PAX	1	2.4.2.30	NAD(+)ADP-ribosyltransferase
proteindisulfideisomerase,putative	2DMM	A	5.3.4.1	Proteindisulfide-isomerase
serine/threonine-proteinkinaseA,putative,proteinkinase,putative	2JAV	A	2.7.11.1	Non-specificserine/threonineproteinkinase
superoxidedismutase,putative	1AR4	A	1.15.1.1	Superoxidedismutase
3,2-trans-enoyl-CoAisomerase,mitochondrialprecursor,putative	1SG4	C	5.3.3.8	Dodecenoyl-CoAisomerase
superoxidedismutase,putative	1XRE	B	1.15.1.1	Superoxidedismutase
tryptophanyl-tRNAsynthetase,putative	1O5T	A	6.1.1.2	Tryptophan--tRNAligase
2-oxoglutaratedehydrogenase,E2component,dihydrolipoamidesuccinyltransferase,putative	1SCZ	A	2.3.1.61	Dihydrolipoyllysine-residuesuccinyltransferase
ubiquitin-conjugatingenzymeE2,putative	2AAK	1	6.3.2.19	Ubiquitin--proteinligase
serine/threonineproteinphosphatase,putative	1U32	A	3.1.3.16	Phosphoproteinphosphatase
glyceraldehyde3-phosphatedehydrogenase,cytosolic,putative	1DC3	B	1.2.1.12	Glyceraldehyde-3-phosphatedehydrogenase(phosphorylating)
DNA-directedRNApolymerasesII,putative	1HMJ	A	2.7.7.6	DNA-directedRNApolymerase
AMPdeaminase,putative	2A3L	A	3.5.4.6	AMPdeaminase
ubiquitinhydrolase,putative,cysteinepeptidase,ClanCA,familyC19,putative	2F1Z	B	3.1.2.15	Ubiquitinthiolesterase
mitochondrialDNApolymerasebeta-PAK,putative	1RPL	1	2.7.7.7	DNA-directedDNApolymerase
dihydrolipoamideacetyltransferase,putative	1Y8P	B	2.3.1.12	Dihydrolipoyllysine-residueacetyltransferase
DNA-directedRNApolymerasesII,putative	1HMJ	A	2.7.7.6	DNA-directedRNApolymerase
proteinkinase,putative	1H4L	B	2.7.11.22	Cyclin-dependentkinase
glutaminylcyclase,putative	2AFZ	B	2.3.2.5	Glutaminyl-peptidecyclotransferase
alanyl-tRNAsynthetase,putative	1YGB	A	6.1.1.7	Alanine--tRNAligase
serine/threonine-proteinphosphatase2A,catalyticsubunit,putative	1U32	A	3.1.3.16	Phosphoproteinphosphatase
AMPdeaminase,putative	2A3L	A	3.5.4.6	AMPdeaminase
tryptophanyl-tRNAsynthetase,putative	1O5T	A	6.1.1.2	Tryptophan--tRNAligase
metalloprotease-likeprotein,putative,peptidedeformylase,putative,polypeptidedeformylase,putative	1N5N	A	3.5.1.88	Peptidedeformylase
ubiquitinhydrolase,putative,cysteinepeptidase,ClanCA,familyC19,putative	2F1Z	B	3.1.2.15	Ubiquitinthiolesterase
vesicle-fusingATPase,putative,vesicular-fusionproteinNSF,putative,N-ethylmaleimidesensitivefusionprotein,putative	1NSF	1	3.6.4.6	Vesicle-fusingATPase
metalloprotease-likeprotein,putative,peptidedeformylase,putative,polypeptidedeformylase,putative	1N5N	A	3.5.1.88	Peptidedeformylase

Matching Annotation	Homologous	7	Trypanosoma_cruzi|AAHK01000781|Tc00.1047053506627.10|Annotation|GenBank|(proteincoding)(441letters)
Matching Annotation	Homologous	7	Trypanosoma_cruzi|AAHK01000897|Tc00.1047053506863.10|Annotation|GenBank|(proteincoding)(959letters)
Matching Annotation	Homologous	7	Trypanosoma_cruzi|AAHK01000969|Tc00.1047053506477.20|Annotation|GenBank|(proteincoding)(726letters)
Matching Annotation	Homologous	7	Trypanosoma_cruzi|AAHK01001631|Tc00.1047053506419.20|Annotation|GenBank|(proteincoding)(680letters)
Matching Annotation	Homologous	7	Trypanosoma_cruzi|AAHK01001659|Tc00.1047053511235.20|Annotation|GenBank|(proteincoding)(214letters)
Matching Annotation	Homologous	7	Trypanosoma_cruzi|AAHK01001706|Tc00.1047053506851.10|Annotation|GenBank|(proteincoding)(763letters)
Matching Annotation	Homologous	7	Trypanosoma_cruzi|AAHK01002091|Tc00.1047053510597.9|Annotation|GenBank|(proteincoding)(695letters)
Matching Annotation	Homologous	7	Trypanosoma_cruzi|AAHK01002627|Tc00.1047053510905.19|Annotation|GenBank|(proteincoding)(246letters)
Matching Annotation	Homologous	7	Trypanosoma_cruzi|AAHK01002638|Tc00.1047053510443.9|Annotation|GenBank|(proteincoding)(413letters)
Matching Annotation	Analogous	4	Trypanosoma_cruzi|AAHK01000390|Tc00.1047053509941.100|Annotation|GenBank|(proteincoding)(693letters)
Matching Annotation	Analogous	5	Trypanosoma_cruzi|AAHK01000085|Tc00.1047053510303.210|Annotation|GenBank|(proteincoding)(717letters)
Matching Annotation	Specific of T. cruzi	1	Trypanosoma_cruzi|AAHK01002040|Tc00.1047053503555.30|Annotation|GenBank|(proteincoding)(492letters)
Matching Annotation	Specific of T. cruzi	3	Trypanosoma_cruzi|AAHK01001930|Tc00.1047053508595.50|Annotation|GenBank|(proteincoding)(181letters)
Matching Annotation	Specific of T. cruzi	3	Trypanosoma_cruzi|AAHK01002533|Tc00.1047053507297.10|Annotation|GenBank|(proteincoding)(247letters)
Matching Annotation	Specific of T. cruzi	3	Trypanosoma_cruzi|AAHK01003274|Tc00.1047053504507.5|Annotation|GenBank|(proteincoding)(388letters)
Matching Annotation	Specific of T. cruzi	4	Trypanosoma_cruzi|AAHK01002082|Tc00.1047053507165.50|Annotation|GenBank|(proteincoding)(332letters)
Matching Annotation	Specific of T. cruzi	4	Trypanosoma_cruzi|AAHK01002160|Tc00.1047053507793.20|Annotation|GenBank|(proteincoding)(332letters)
Matching Annotation	Specific of T. cruzi	5	Trypanosoma_cruzi|AAHK01000588|Tc00.1047053506649.20|Annotation|GenBank|(proteincoding)(898letters)
Matching Annotation	Specific of T. cruzi	6	Trypanosoma_cruzi|AAHK01000003|Tc00.1047053506529.550|Annotation|GenBank|(proteincoding)(322letters)
Matching Annotation	Specific of T. cruzi	6	Trypanosoma_cruzi|AAHK01001793|Tc00.1047053507537.20|Annotation|GenBank|(proteincoding)(426letters)
Matching Annotation	Specific of T. cruzi	6	Trypanosoma_cruzi|AAHK01002523|Tc00.1047053505763.19|Annotation|GenBank|(proteincoding)(646letters)
Matching Annotation	Specific of T. cruzi	7	Trypanosoma_cruzi|AAHK01000060|Tc00.1047053509429.320|Annotation|GenBank|(proteincoding)(467letters)
Matching Annotation	Specific of T. cruzi	7	Trypanosoma_cruzi|AAHK01000135|Tc00.1047053507603.260|Annotation|GenBank|(proteincoding)(467letters)
Matching Annotation	Specific of T. cruzi	7	Trypanosoma_cruzi|AAHK01000135|Tc00.1047053507603.270|Annotation|GenBank|(proteincoding)(467letters)
Matching Annotation	Specific of T. cruzi	7	Trypanosoma_cruzi|AAHK01003234|Tc00.1047053509401.30|Annotation|GenBank|(proteincoding)(467letters)
Matching Annotation	Conflicting Clustering (a) (Homologous/Analogous)	7	Trypanosoma_cruzi|AAHK01001379|Tc00.1047053511895.10|Annotation|GenBank|(proteincoding)(499letters)
Matching Annotation	Conflicting Clustering (a) (Homologous/Analogous)	7	Trypanosoma_cruzi|AAHK01002576|Tc00.1047053510581.20|Annotation|GenBank|(proteincoding)(502letters)
Matching Annotation	Conflicting Clustering (a) (Homologous/Analogous)	7	Trypanosoma_cruzi|AAHK01002962|Tc00.1047053509449.10|Annotation|GenBank|(proteincoding)(473letters)
Surface	Homologous	4	Trypanosoma_cruzi|AAHK01000311|Tc00.1047053510503.100|Annotation|GenBank|(proteincoding)(672letters)
Surface	Homologous	4	Trypanosoma_cruzi|AAHK01000647|Tc00.1047053509369.50|Annotation|GenBank|(proteincoding)(443letters)
Surface	Homologous	4	Trypanosoma_cruzi|AAHK01002494|Tc00.1047053410797.10|Annotation|GenBank|(proteincoding)(543letters)
Surface	Homologous	6	Trypanosoma_cruzi|AAHK01000047|Tc00.1047053508165.310|Annotation|GenBank|(proteincoding)(725letters)
Surface	Homologous	6	Trypanosoma_cruzi|AAHK01000136|Tc00.1047053506289.140|Annotation|GenBank|(proteincoding)(818letters)
Surface	Homologous	6	Trypanosoma_cruzi|AAHK01000136|Tc00.1047053506289.170|Annotation|GenBank|(proteincoding)(708letters)
Surface	Homologous	6	Trypanosoma_cruzi|AAHK01000136|Tc00.1047053506289.210|Annotation|GenBank|(proteincoding)(733letters)
Surface	Homologous	6	Trypanosoma_cruzi|AAHK01000505|Tc00.1047053511257.100|Annotation|GenBank|(proteincoding)(786letters)
Surface	Homologous	6	Trypanosoma_cruzi|AAHK01000667|Tc00.1047053511151.54|Annotation|GenBank|(proteincoding)(725letters)
Surface	Homologous	6	Trypanosoma_cruzi|AAHK01002593|Tc00.1047053506515.29|Annotation|GenBank|(proteincoding)(272letters)
Surface	Homologous	6	Trypanosoma_cruzi|AAHK01002659|Tc00.1047053509483.10|Annotation|GenBank|(proteincoding)(322letters)
Surface	Specific of T. cruzi	4	Trypanosoma_cruzi|AAHK01000184|Tc00.1047053511211.90|Annotation|GenBank|(proteincoding)(567letters)
Surface	Specific of T. cruzi	4	Trypanosoma_cruzi|AAHK01000279|Tc00.1047053510565.150|Annotation|GenBank|(proteincoding)(589letters)
Surface	Specific of T. cruzi	4	Trypanosoma_cruzi|AAHK01000551|Tc00.1047053507623.110|Annotation|GenBank|(proteincoding)(568letters)
Surface	Specific of T. cruzi	4	Trypanosoma_cruzi|AAHK01000586|Tc00.1047053508699.100|Annotation|GenBank|(proteincoding)(566letters)
Surface	Specific of T. cruzi	4	Trypanosoma_cruzi|AAHK01000586|Tc00.1047053508699.90|Annotation|GenBank|(proteincoding)(621letters)
Surface	Specific of T. cruzi	4	Trypanosoma_cruzi|AAHK01000598|Tc00.1047053509011.80|Annotation|GenBank|(proteincoding)(592letters)
Surface	Specific of T. cruzi	4	Trypanosoma_cruzi|AAHK01000802|Tc00.1047053506587.100|Annotation|GenBank|(proteincoding)(566letters)
Surface	Specific of T. cruzi	4	Trypanosoma_cruzi|AAHK01000883|Tc00.1047053509205.100|Annotation|GenBank|(proteincoding)(567letters)
Surface	Specific of T. cruzi	4	Trypanosoma_cruzi|AAHK01000898|Tc00.1047053506163.10|Annotation|GenBank|(proteincoding)(567letters)
Surface	Specific of T. cruzi	4	Trypanosoma_cruzi|AAHK01000898|Tc00.1047053506163.20|Annotation|GenBank|(proteincoding)(567letters)
Surface	Specific of T. cruzi	4	Trypanosoma_cruzi|AAHK01000907|Tc00.1047053508813.40|Annotation|GenBank|(proteincoding)(567letters)
Surface	Specific of T. cruzi	4	Trypanosoma_cruzi|AAHK01000994|Tc00.1047053505965.10|Annotation|GenBank|(proteincoding)(567letters)

AMPdeaminase2,putative	2A3L	A	3.5.4.6	AMPdeaminase
alanyl-tRNAsynthetase,putative	1YGB	A	6.1.1.7	Alanine--tRNAligase
vesicle-fusingATPase,putative,vesicular-fusionproteinNSF,putative,N-ethylmaleimidesensitivefusionprotein,putative	1NSF	1	3.6.4.6	Vesicle-fusingATPase
proteinkinase,putative	2EUE	B	2.7.11.1	Non-specificserine/threonineproteinkinase
glyceraldehyde3-phosphatedehydrogenase,cytosolic,putative	1DC3	B	1.2.1.12	Glyceraldehyde-3-phosphatedehydrogenase(phosphorylating)
proteinkinase,putative,serine/threonine-proteinkinasenek1,putative	2JAV	A	2.7.11.1	Non-specificserine/threonineproteinkinase
proteinkinase,putative	2EUE	B	2.7.11.1	Non-specificserine/threonineproteinkinase
tryptophanyl-tRNAsynthetase,putative	1O5T	A	6.1.1.2	Tryptophan--tRNAligase
valyl-tRNAsynthetase,putative	1WKA	A	6.1.1.9	Valine--tRNAligase
2,4-dienoyl-coareductaseFADH1,putative	1PS9	A	1.3.1.34	2,4-dienoyl-CoAreductase(NADPH)
2,4-dienoyl-coareductase-likeprotein,putative	1PS9	A	1.3.1.34	2,4-dienoyl-CoAreductase(NADPH)
trypanothionereductase,putative	1GXF	B	1.8.1.12	Trypanothione-disulfidereductase
cysteinepeptidase,putative,cathepsinL-like,putative,majorcysteineproteinase,putative,cysteinepeptidase,clanCA,familyC1,putative	2AIM	1	3.4.22.51	Cruzipain
cruzipainprecursor,putative,cathepsinL-like,putative,majorcysteineproteinase,putative	2AIM	1	3.4.22.51	Cruzipain
trypanothionereductase,putative	1GXF	B	1.8.1.12	Trypanothione-disulfidereductase
cysteinesynthase,putative	1Z7W	A	2.5.1.47	Cysteinesynthase
cysteinesynthase,putative	1Z7W	A	2.5.1.47	Cysteinesynthase
P-typeH+-ATPase,putative	1MHS	B	3.6.3.6	Proton-exportingATPase
cysteinepeptidase,putative,cysteinepeptidase,clanCA,familyC1,cathepsinL-like,putative	2AIM	1	3.4.22.51	Cruzipain
cysteinepeptidase,putative,cysteinepeptidase,clanCA,familyC1,cathepsinL-like,putative	2AIM	1	3.4.22.51	Cruzipain
P-typeH+-ATPase,putative	1MHS	B	3.6.3.6	Proton-exportingATPase
cysteinepeptidase,putative,cysteinepeptidase,clanCA,familyC1,cathepsinL-like,putative	2AIM	1	3.4.22.51	Cruzipain
cysteinepeptidase,putative,cysteinepeptidase,clanCA,familyC1,cathepsinL-like,putative	2AIM	1	3.4.22.51	Cruzipain
cruzipainprecursor,putative,cathepsinL-like,putative,majorcysteineproteinase,putative,cysteinepeptidase,clanCA,familyC1,putative	2AIM	1	3.4.22.51	Cruzipain
cysteinepeptidase,putative,cysteinepeptidase,clanCA,familyC1,cathepsinL-like,putative	2AIM	1	3.4.22.51	Cruzipain
receptor-typeadenylatecyclase,putative	1FX2	A	4.6.1.1	Adenylatecyclase
receptor-typeadenylatecyclase,putative	1FX2	A	4.6.1.1	Adenylatecyclase
receptor-typeadenylatecyclase,putative	1FX2	A	4.6.1.1	Adenylatecyclase
surfaceproteaseGP63,putative	1LML	1	3.4.24.36	Leishmanolysin
surfaceproteaseGP63,putative	1LML	1	3.4.24.36	Leishmanolysin
surfaceproteaseGP63,putative,metallopeptidase,putative	1LML	1	3.4.24.36	Leishmanolysin
surfaceproteaseGP63,putative	1LML	1	3.4.24.36	Leishmanolysin
surfaceproteaseGP63,putative	1LML	1	3.4.24.36	Leishmanolysin
surfaceproteaseGP63,putative	1LML	1	3.4.24.36	Leishmanolysin
surfaceproteaseGP63,putative	1LML	1	3.4.24.36	Leishmanolysin
surfaceproteaseGP63,putative	1LML	1	3.4.24.36	Leishmanolysin
surfaceproteaseGP63,putative	1LML	1	3.4.24.36	Leishmanolysin
trans-sialidase,putative	3SIL	1	3.2.1.18	Exo-alpha-sialidase
surfaceproteaseGP63,putative	1LML	1	3.4.24.36	Leishmanolysin
surfaceproteaseGP63,putative	1LML	1	3.4.24.36	Leishmanolysin
surfaceproteaseGP63,putative	1LML	1	3.4.24.36	Leishmanolysin
surfaceproteaseGP63,putative	1LML	1	3.4.24.36	Leishmanolysin
surfaceproteaseGP63,putative	1LML	1	3.4.24.36	Leishmanolysin
surfaceproteaseGP63,putative	1LML	1	3.4.24.36	Leishmanolysin
surfaceproteaseGP63,putative	1LML	1	3.4.24.36	Leishmanolysin
surfaceproteaseGP63,putative	1LML	1	3.4.24.36	Leishmanolysin
surfaceproteaseGP63,putative	1LML	1	3.4.24.36	Leishmanolysin
surfaceproteaseGP63,putative	1LML	1	3.4.24.36	Leishmanolysin
surfaceproteaseGP63,putative	1LML	1	3.4.24.36	Leishmanolysin
surfaceproteaseGP63,putative	1LML	1	3.4.24.36	Leishmanolysin
surfaceproteaseGP63,putative	1LML	1	3.4.24.36	Leishmanolysin

Surface	Specific of T. cruzi	4	Trypanosoma_cruzi|AAHK01001085|Tc00.1047053506257.50|Annotation|GenBank|(proteincoding)(600letters)
Surface	Specific of T. cruzi	4	Trypanosoma_cruzi|AAHK01001439|Tc00.1047053510899.10|Annotation|GenBank|(proteincoding)(566letters)
Surface	Specific of T. cruzi	4	Trypanosoma_cruzi|AAHK01001639|Tc00.1047053505931.10|Annotation|GenBank|(proteincoding)(516letters)
Surface	Specific of T. cruzi	4	Trypanosoma_cruzi|AAHK01001639|Tc00.1047053505931.20|Annotation|GenBank|(proteincoding)(579letters)
Surface	Specific of T. cruzi	4	Trypanosoma_cruzi|AAHK01001927|Tc00.1047053511203.10|Annotation|GenBank|(proteincoding)(566letters)
Surface	Specific of T. cruzi	4	Trypanosoma_cruzi|AAHK01002370|Tc00.1047053504397.20|Annotation|GenBank|(proteincoding)(567letters)
Surface	Specific of T. cruzi	4	Trypanosoma_cruzi|AAHK01002462|Tc00.1047053506921.10|Annotation|GenBank|(proteincoding)(622letters)
Surface	Specific of T. cruzi	4	Trypanosoma_cruzi|AAHK01002508|Tc00.1047053508475.30|Annotation|GenBank|(proteincoding)(566letters)
Surface	Specific of T. cruzi	4	Trypanosoma_cruzi|AAHK01002632|Tc00.1047053505615.10|Annotation|GenBank|(proteincoding)(592letters)
Surface	Specific of T. cruzi	4	Trypanosoma_cruzi|AAHK01002639|Tc00.1047053508825.10|Annotation|GenBank|(proteincoding)(565letters)
Surface	Specific of T. cruzi	4	Trypanosoma_cruzi|AAHK01003293|Tc00.1047053510873.20|Annotation|GenBank|(proteincoding)(565letters)
Surface	Specific of T. cruzi	6	Trypanosoma_cruzi|AAHK01002308|Tc00.1047053507197.10|Annotation|GenBank|(proteincoding)(697letters)
Surface	Conflicting Clustering (a) (Homologous/Specific of T. cruzi)	4	Trypanosoma_cruzi|AAHK01000005|Tc00.1047053511277.610|Annotation|GenBank|(proteincoding)(514letters)
Surface	Conflicting Clustering (a) (Homologous/Specific of T. cruzi)	4	Trypanosoma_cruzi|AAHK01000020|Tc00.1047053506401.380|Annotation|GenBank|(proteincoding)(543letters)
Surface	Conflicting Clustering (a) (Homologous/Specific of T. cruzi)	4	Trypanosoma_cruzi|AAHK01000028|Tc00.1047053506435.370|Annotation|GenBank|(proteincoding)(598letters)
Surface	Conflicting Clustering (a) (Homologous/Specific of T. cruzi)	4	Trypanosoma_cruzi|AAHK01000043|Tc00.1047053507993.350|Annotation|GenBank|(proteincoding)(543letters)
Surface	Conflicting Clustering (a) (Homologous/Specific of T. cruzi)	4	Trypanosoma_cruzi|AAHK01000064|Tc00.1047053508999.170|Annotation|GenBank|(proteincoding)(531letters)
Surface	Conflicting Clustering (a) (Homologous/Specific of T. cruzi)	4	Trypanosoma_cruzi|AAHK01000094|Tc00.1047053506321.240|Annotation|GenBank|(proteincoding)(544letters)
Surface	Conflicting Clustering (a) (Homologous/Specific of T. cruzi)	4	Trypanosoma_cruzi|AAHK01000181|Tc00.1047053508693.100|Annotation|GenBank|(proteincoding)(543letters)
Surface	Conflicting Clustering (a) (Homologous/Specific of T. cruzi)	4	Trypanosoma_cruzi|AAHK01000219|Tc00.1047053510657.200|Annotation|GenBank|(proteincoding)(543letters)
Surface	Conflicting Clustering (a) (Homologous/Specific of T. cruzi)	4	Trypanosoma_cruzi|AAHK01000354|Tc00.1047053510747.40|Annotation|GenBank|(proteincoding)(542letters)
Surface	Conflicting Clustering (a) (Homologous/Specific of T. cruzi)	4	Trypanosoma_cruzi|AAHK01000505|Tc00.1047053511257.60|Annotation|GenBank|(proteincoding)(543letters)
Surface	Conflicting Clustering (a) (Homologous/Specific of T. cruzi)	4	Trypanosoma_cruzi|AAHK01000520|Tc00.1047053510761.80|Annotation|GenBank|(proteincoding)(542letters)
Surface	Conflicting Clustering (a) (Homologous/Specific of T. cruzi)	4	Trypanosoma_cruzi|AAHK01000757|Tc00.1047053511281.50|Annotation|GenBank|(proteincoding)(543letters)
Surface	Conflicting Clustering (a) (Homologous/Specific of T. cruzi)	4	Trypanosoma_cruzi|AAHK01000974|Tc00.1047053505989.70|Annotation|GenBank|(proteincoding)(543letters)
Surface	Conflicting Clustering (a) (Homologous/Specific of T. cruzi)	4	Trypanosoma_cruzi|AAHK01001466|Tc00.1047053508545.40|Annotation|GenBank|(proteincoding)(580letters)
Surface	Conflicting Clustering (a) (Homologous/Specific of T. cruzi)	4	Trypanosoma_cruzi|AAHK01001717|Tc00.1047053506867.40|Annotation|GenBank|(proteincoding)(567letters)
Surface	Conflicting Clustering (a) (Homologous/Specific of T. cruzi)	4	Trypanosoma_cruzi|AAHK01002140|Tc00.1047053510263.30|Annotation|GenBank|(proteincoding)(543letters)
Surface	Conflicting Clustering (a) (Homologous/Specific of T. cruzi)	4	Trypanosoma_cruzi|AAHK01002432|Tc00.1047053508609.10|Annotation|GenBank|(proteincoding)(543letters)
Surface	Conflicting Clustering (a) (Homologous/Specific of T. cruzi)	4	Trypanosoma_cruzi|AAHK01002568|Tc00.1047053508611.30|Annotation|GenBank|(proteincoding)(543letters)
Surface	Conflicting Clustering (a) (Homologous/Specific of T. cruzi)	4	Trypanosoma_cruzi|AAHK01002733|Tc00.1047053511723.10|Annotation|GenBank|(proteincoding)(543letters)
Surface	Conflicting Clustering (a) (Homologous/Specific of T. cruzi)	4	Trypanosoma_cruzi|AAHK01002752|Tc00.1047053507919.10|Annotation|GenBank|(proteincoding)(580letters)
Surface	Conflicting Clustering (a) (Homologous/Specific of T. cruzi)	4	Trypanosoma_cruzi|AAHK01002825|Tc00.1047053511035.10|Annotation|GenBank|(proteincoding)(544letters)
Surface	Conflicting Clustering (a) (Homologous/Specific of T. cruzi)	4	Trypanosoma_cruzi|AAHK01003120|Tc00.1047053507917.10|Annotation|GenBank|(proteincoding)(543letters)
Hypothetical	Homologous	5	Trypanosoma_cruzi|AAHK01001589|Tc00.1047053507057.20|Annotation|GenBank|(proteincoding)(260letters)
Hypothetical	Homologous	6	Trypanosoma_cruzi|AAHK01000085|Tc00.1047053510303.230|Annotation|GenBank|(proteincoding)(200letters)
Hypothetical	Homologous	6	Trypanosoma_cruzi|AAHK01003248|Tc00.1047053511763.19|Annotation|GenBank|(proteincoding)(445letters)
Hypothetical	Homologous	7	Trypanosoma_cruzi|AAHK01000484|Tc00.1047053509207.130|Annotation|GenBank|(proteincoding)(421letters)
Hypothetical	Homologous	7	Trypanosoma_cruzi|AAHK01000774|Tc00.1047053506857.60|Annotation|GenBank|(proteincoding)(422letters)
Hypothetical	Analogous	6	Trypanosoma_cruzi|AAHK01001147|Tc00.1047053509005.50|Annotation|GenBank|(proteincoding)(521letters)
Conflicting Annotation (b)	Homologous	5	Trypanosoma_cruzi|AAHK01000447|Tc00.1047053506959.70|Annotation|GenBank|(proteincoding)(560letters)
Conflicting Annotation (b)	Homologous	5	Trypanosoma_cruzi|AAHK01000821|Tc00.1047053503453.100|Annotation|GenBank|(proteincoding)(560letters)

MHOLline Quality	Identity	Coverage
1. Very High	>=75%	>=90%
2. High	>=50% and <75%	>=90%
3. Good	>=50%	>=70% and <90%
4. Medium to Good	>=35% and <50%	>=70%
5. Medium to Low	>=25% and <35%	>=70%
6. Low	>=25%	>=50% and <70%
7. Very Low	>=25%	>=30% and <50%

(a) Conflicting clustering between results obtained by KEGG and Swiss-Prot databases using AnEnPi methodology.

(b) Conflicting annotation between the methodology proposed in this work and GeneDB.

surfaceproteaseGP63,putative	1LML	1	3.4.24.36	Leishmanolysin
surfaceproteaseGP63,putative	1LML	1	3.4.24.36	Leishmanolysin
surfaceproteaseGP63,putative	1LML	1	3.4.24.36	Leishmanolysin
surfaceproteaseGP63,putative	1LML	1	3.4.24.36	Leishmanolysin
surfaceproteaseGP63,putative	1LML	1	3.4.24.36	Leishmanolysin
surfaceproteaseGP63,putative	1LML	1	3.4.24.36	Leishmanolysin
surfaceproteaseGP63,putative	1LML	1	3.4.24.36	Leishmanolysin
surfaceproteaseGP63,putative	1LML	1	3.4.24.36	Leishmanolysin
surfaceproteaseGP63,putative	1LML	1	3.4.24.36	Leishmanolysin
surfaceproteaseGP63,putative	1LML	1	3.4.24.36	Leishmanolysin
surfaceproteaseGP63,putative	1LML	1	3.4.24.36	Leishmanolysin
surfaceproteaseGP63,putative	1LML	1	3.4.24.36	Leishmanolysin
surfaceproteaseGP63,putative,metallopeptidase,putative	1LML	1	3.4.24.36	Leishmanolysin
surfaceproteaseGP63,putative	1LML	1	3.4.24.36	Leishmanolysin
surfaceproteaseGP63,putative,metallopeptidase,putative	1LML	1	3.4.24.36	Leishmanolysin
surfaceproteaseGP63,putative,metallopeptidase,putative	1LML	1	3.4.24.36	Leishmanolysin
surfaceproteaseGP63,putative,metallopeptidase,putative	1LML	1	3.4.24.36	Leishmanolysin
surfaceproteaseGP63,putative,metallopeptidase,putative	1LML	1	3.4.24.36	Leishmanolysin
surfaceproteaseGP63,putative,metallopeptidase,putative	1LML	1	3.4.24.36	Leishmanolysin
surfaceproteaseGP63,putative,metallopeptidase,putative	1LML	1	3.4.24.36	Leishmanolysin
surfaceproteaseGP63,putative,metallopeptidase,putative	1LML	1	3.4.24.36	Leishmanolysin
surfaceproteaseGP63,putative,metallopeptidase,putative	1LML	1	3.4.24.36	Leishmanolysin
surfaceproteaseGP63,putative,metallopeptidase,putative	1LML	1	3.4.24.36	Leishmanolysin
surfaceproteaseGP63,putative,metallopeptidase,putative	1LML	1	3.4.24.36	Leishmanolysin
surfaceproteaseGP63,putative,metallopeptidase,putative	1LML	1	3.4.24.36	Leishmanolysin
surfaceproteaseGP63,putative	1LML	1	3.4.24.36	Leishmanolysin
surfaceproteaseGP63,putative	1LML	1	3.4.24.36	Leishmanolysin
surfaceproteaseGP63,putative,metallopeptidase,putative	1LML	1	3.4.24.36	Leishmanolysin
surfaceproteaseGP63,putative	1LML	1	3.4.24.36	Leishmanolysin
surfaceproteaseGP63,putative	1LML	1	3.4.24.36	Leishmanolysin
surfaceproteaseGP63,putative,metallopeptidase,putative	1LML	1	3.4.24.36	Leishmanolysin
surfaceproteaseGP63,putative	1LML	1	3.4.24.36	Leishmanolysin
surfaceproteaseGP63,putative,metallopeptidase,putative	1LML	1	3.4.24.36	Leishmanolysin
surfaceproteaseGP63,putative	1LML	1	3.4.24.36	Leishmanolysin
hypotheticalprotein,conserved	1ZAK	B	2.7.4.3	Adenylatekinase
hypotheticalprotein,conserved	1RZW	A	3.1.1.29	Aminoacyl-tRNAhydrolase
hypotheticalprotein,conserved	1NDI	B	2.3.1.7	CarnitineO-acetyltransferase
hypotheticalprotein,conserved	1F8A	B	5.2.1.8	Peptidylprolylisomerase
hypotheticalprotein,conserved	1F8A	B	5.2.1.8	Peptidylprolylisomerase
hypotheticalprotein,conserved	1TGL	1	3.1.1.3	Triacylglycerollipase
serinepalmitoyltransferase,putative	1FC4	B	2.3.1.29	GlycineC-acetyltransferase
serinepalmitoyltransferase,putative	1FC4	B	2.3.1.29	GlycineC-acetyltransferase
